# A Gamified Personalized Normative Feedback App to Reduce Drinking Among Sexual Minority Women: Randomized Controlled Trial and Feasibility Study

**DOI:** 10.2196/34853

**Published:** 2022-05-13

**Authors:** Sarah C Boyle, Joseph W LaBrie, Bradley M Trager, Lauren D Costine

**Affiliations:** 1 HeadsUp Labs Department of Psychology Loyola Marymount University Los Angeles, CA United States; 2 Awaken Therapy Center Los Angeles, CA United States

**Keywords:** sexual minority women, alcohol, intervention, social norms, gamification, mobile phone, smartphone

## Abstract

**Background:**

Sexual minority women disproportionately engage in heavy drinking and shoulder the burden of alcohol dependence. Although several intensive interventions are being developed to meet the needs of treatment-seeking sexual minority women, there remains a lack of preventive interventions to reduce drinking and its consequences among women not yet motivated to reduce their alcohol consumption.

**Objective:**

We aimed to examine the feasibility and efficacy of reducing alcohol-related risks via personalized normative feedback (PNF) on alcohol use and coping delivered within LezParlay, a social media–inspired digital competition designed to challenge negative stereotypes about lesbian, bisexual, and queer (LBQ)–identified sexual minority women.

**Methods:**

Feasibility was assessed by examining engagement with LezParlay outside the context of an incentivized research study, assessing the characteristics of the LBQ women taking part, and examining the competition’s ability to derive risk-reducing actual norms as well as levels of acceptability and perceived benefits reported by participants. Intervention efficacy was examined by randomizing a subsample of 499 LBQ alcohol consumers (ie, drinkers) already taking part in the competition to receive sexual identity–specific PNF on alcohol use and coping, alcohol use only, or control topics over only 2 rounds of play. Changes in alcohol use and negative consequences were examined 2 and 4 months after the delivery of treatment PNF.

**Results:**

A total of 2667 diverse LBQ women played ≥1 round of LezParlay. The competition attracted large numbers of moderate and heavy drinkers; however, risk-reducing actual norms could still be derived from competition rounds and featured in PNF. Efficacy results revealed that drinkers who received PNF on alcohol use and both alcohol use and coping had similar reductions in their weekly drinks (*P*=.003; *P*<.001), peak drinks (*P*<.001; *P*<.001), and negative consequences (*P*<.001; *P*<.001) relative to those who received PNF on control topics at the 2-month follow-up. However, at the 4-month follow-up, reductions in alcohol consumption outcomes faded among those who received alcohol PNF only (weekly: *P*=.06; peak: *P*=.11), whereas they remained relatively robust among those who received PNF on both alcohol use and coping (weekly: *P*=.02; peak: *P*=.03). Finally, participants found the competition highly acceptable and psychologically beneficial as a whole.

**Conclusions:**

The LezParlay competition was found to be a feasible and efficacious means of reducing alcohol-related risks in this population. Our findings demonstrate the utility of correcting sexual identity–specific drinking and coping norms to reduce alcohol-related risks among LBQ women and suggest that this approach may also prove fruitful in other stigmatized health disparity populations. To engage these populations in the real world and expand the psychological benefits associated with PNF, our findings also point to packaging PNF within a broader, culturally tailored competition designed to challenge negative group stereotypes.

**Trial Registration:**

ClinicalTrials.gov NCT03884478; https://clinicaltrials.gov/ct2/show/NCT03884478

**International Registered Report Identifier (IRRID):**

RR2-10.2196/24647

## Introduction

### Background

Relative to women who identify as heterosexual, experience only opposite-sex attractions, and only have sex with men, research has reliably documented more frequent and intense alcohol consumption [1-3], as well as a greater likelihood of negative alcohol-related consequences and alcohol dependence [2-4], among sexual minority women, a population that includes women who psychologically identify as lesbian, bisexual, or queer (LBQ), in addition to those who report having sex with women and experiencing same-sex attraction [5,6]. Although several culturally tailored interventions are currently being developed to meet the needs of heavy drinking sexual minority women seeking treatment for alcohol use disorder (AUD) [7,8], to date, there remains a lack of preventive, culturally tailored interventions to reduce alcohol-related harm among alcohol-consuming sexual minority women *who are not yet* motivated to reduce their drinking. Seeking to address this void, this study evaluates the degree to which an evidence-based personalized normative feedback (PNF) intervention embedded within a culturally tailored digital competition can engage LBQ-identified sexual minority women and reduce their alcohol-related risks.

### Antecedents to Heavy Drinking Among Sexual Minority Women and Targets for Intervention

Consistent with the sexual minority stress model [9], extant research has linked greater alcohol consumption and negative consequences among sexual minority women to the internalization of sexual minority stigma [10-12] and experiences of harassment, discrimination, and violence due to sexual minority status [13-15]. These findings have informed the recent development of 2 stigma-coping–focused digital programs designed for heavy drinking sexual minority women seeking treatment for AUD only [7] and both AUD and poor mental health [8]. Although these programs hold promise for sexual minority women motivated to seek help, they appear unlikely to attract or engage the larger population of sexual minority women who do not view their mental health or drinking as problematic.

Recent research suggests that to motivate reductions in drinking among those not seeking treatment, it may be beneficial to target the elevated perceptions of sexual identity–specific drinking norms [16-20], which appear to be a consequence of the central role that alcohol use plays in queer socialization contexts [21-24]. Indeed, qualitative accounts from LBQ-identified women suggest that the position of bars and nightclubs as central hubs for queer socialization may lead young LBQ women to view heavy drinking as a normative *rite of passage* [23,24]. Findings from survey studies also suggest that the high visibility of alcohol use in physical and web-based LBQ community spaces may lead LBQ women to perceive heavy drinking as more characteristic or *typical* of LBQ peers than heterosexual women [20]. They tend to substantially overestimate how much and how often LBQ peers drink [16,17,19] and the frequency with which they drink to cope with sexual minority stigma [25].

### Web-Based PNF Interventions

In other heavy drinking populations found to overestimate peer-drinking norms, alcohol-related risks have been reduced through PNF, a brief intervention strategy that only requires members of a social group to answer survey questions about their perceptions of the *typical* group member’s drinking and then report on their own consumption [26-28]. Group members then receive individualized graphical reports highlighting discrepancies between their perceptions of peers’ drinking, peers’ actual drinking, and their own drinking [28,29]. To date, research has yet to investigate whether delivering PNF on LBQ-specific drinking and coping norms is an effective means of reducing alcohol-related risks among LBQ drinkers. However, supporting the promise of PNF for this population, in university and military samples, this strategy has been found particularly effective in reducing alcohol consumption among women [30,31], individuals for whom the reference peer group or community is important to their overall sense of self [32], those reporting coping motivations for drinking [33,34], and heavy drinkers not yet aware that their consumption exceeds normative standards [35].

### Reaching LBQ Drinkers With PNF on Alcohol Use and Stigma Coping

Despite the potential promise of PNF, previous work suggests that LBQ women may comprise a population that is particularly difficult to reach, recruit, and retain in transparent health interventions. For instance, a review of community-based interventions targeting various health risk behaviors in this population identified low response rates, small sample sizes, and problems with attrition as significant challenges to evaluation efforts, reflecting broader difficulties with intervention engagement [36]. Recruitment and engagement concerns are also magnified in the PNF context, as this strategy is most effective in reducing alcohol-related risks among individuals who do not view their drinking as excessive or see themselves as in need of intervention. Moreover, very few PNF interventions have been delivered to populations not attached to institutions or workplaces, and researchers have struggled to implement PNF interventions outside study settings where participation is mandatory or participants are promised compensation at the point of recruitment [37-39].

**Figure 1:**
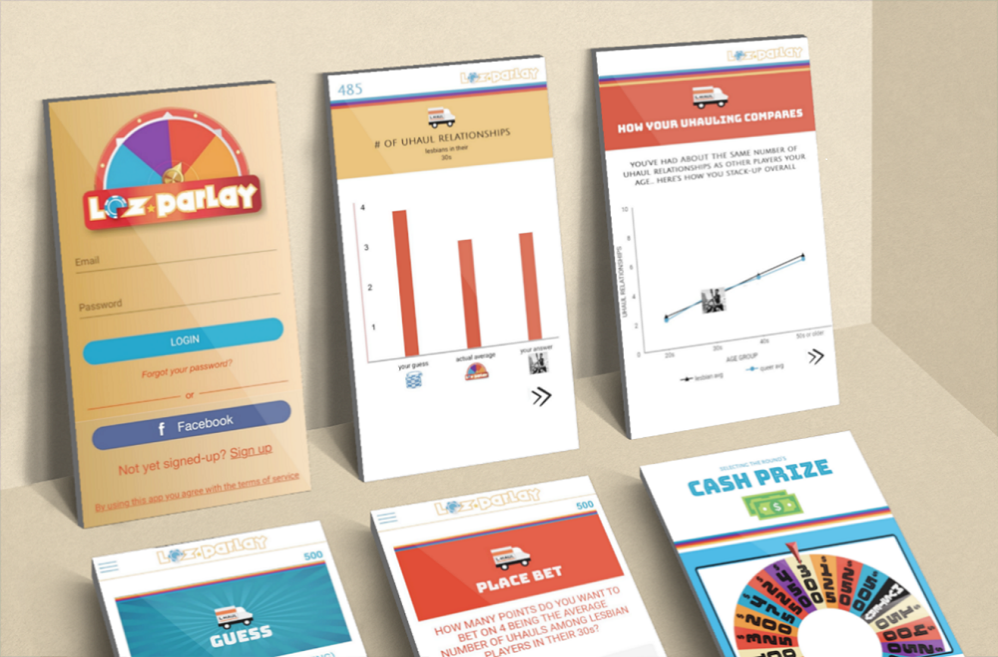
The initial version of LezParlay tested in this trial was a device-responsive HTML5 web application that delivered personalized normative feedback on a number of lesbian, bisexual, and queer stereotypes and health-related topics within the context of a monthly competition.

Seeking to remedy these implementation challenges and extend promising gamified intervention work with college students [40-42], PNF on alcohol use and stigma-coping behaviors was delivered to LBQ drinkers within *LezParlay*, a culturally tailored digital competition designed to challenge negative stereotypes about LBQ women and increase visibility (Figure 1). In brief, the competition comprised 8 monthly rounds wherein LBQ users guessed about the behaviors, attitudes, and experiences of age group and sexual identity–matched peers; wagered points on their guesses being true based on the responses of other users; and reported on their own corresponding behaviors, attitudes, and experiences. At the end of each month, players were SMS text messaged private URLs at which they could view detailed results (ie, PNF) on all or a subset of the round’s questions. All actual norms presented in the detailed results (ie, PNF) were transparently derived from the responses of the players in each subgroup. Users’ scores reflected the accuracy of their LBQ peer perceptions, and each round’s top scorer won a variable cash prize. A complete overview of the digital competition and detailed descriptions of the theory-informed game mechanics and deep-structure cultural adaptations leveraged to bolster appeal and engagement are available in this project’s protocol paper [43].

### This Study

Informed by the Accelerated Creation-to-Sustainment model for the rapid development and evaluation of real-world–ready digital health interventions [44-46], a registered hybrid trial [43] simultaneously examined the degree to which LBQ women would be engaged in the LezParlay competition in the real world (when there was no study framing and participation incentives were not offered) and evaluated whether delivering PNF on alcohol use and stigma coping within the competition would meaningfully reduce alcohol use and negative consequences among participating LBQ drinkers. As shown in Textbox 1, this study examined 5 key questions related to LezParlay’s feasibility and efficacy as an alcohol intervention strategy.

**Key feasibility and efficacy questions addressed in this study.**

**Feasibility**

1. Were lesbian, bisexual, and queer (LBQ) women engaged by the LezParlay competition in the absence of traditional study incentives?
2. Could risk-reducing actual drinking norms be generated in real time from users’ responses to round questions?
3. Did LBQ drinkers taking part in LezParlay find the competition acceptable and psychologically beneficial? What ideas for improvements were submitted?

**Efficacy**

1. Did personalized normative feedback (PNF) designed to correct LBQ peer-drinking norms reduce alcohol-related risks among LBQ drinkers?
2. Did PNF on both LBQ peer-drinking and stigma-coping norms better reduce alcohol-related risks than PNF on LBQ peer-drinking norms alone?

## Methods

### Participants and Procedure

#### Broader Competition

**Figure 2:**
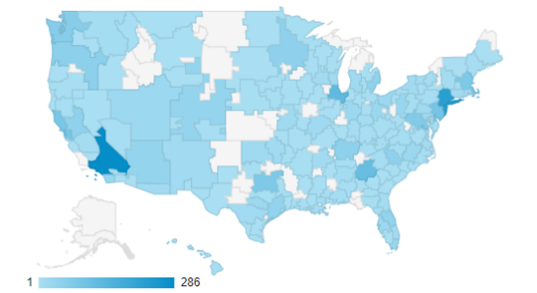
Geodensity of LezParlay users across US metropolitan areas.

LezParlay was advertised to LBQ women as it would be in the real world—as a free, web-based competition designed to test LBQ stereotypes and increase visibility. Despite no traditional study incentives being offered at the point of recruitment or sign-up, 2677 LBQ women took part in the competition between December 2018 and July 2019 and played ≥1 of the 8 monthly rounds. LezParlay’s informational landing page received 4099 unique views during recruitment and competition periods, with digital advertising campaigns responsible for the bulk of these views. Specifically, promotional campaigns on the HER Social app, a popular dating and social networking app for LBQ women, were responsible for 34.01% (1394/4099) of the total landing page visits, whereas campaigns on Facebook or Instagram and Google Search accounted for 32.01% (1312/4099) and 22.98% (942/4099) of the total visits, respectively. Of the 4099 landing page visitors, 2008 (48.99%) advanced directly to create a user account [43] on the LezParlay competition web app. In addition, 669 user accounts were created organically by users who did not view the landing page first but were directly invited to the LezParlay web app by a friend taking part in the competition. Figure 2 provides a visual breakdown of LezParlay users by US metropolitan area, and Table 1 presents basic user characteristics.

**Table 1:** Characteristics of LezParlay users (N=2667).

Characteristic		Participants, n (%)
**Sexual identity**		
	Lesbian	1446 (54.22)
	Bisexual	669 (25.08)
	Queer	562 (21.07)
**Age group (years)**		
	<18	107 (4.01)
	18-24	401 (15.04)
	25-34	1284 (48.14)
	35-44	562 (21.07)
	54-65	240 (9)
	≥66	80 (3)
**Relationship status**		
	Single	1205 (45.18)
	In a relationship	857 (32.13)
	Married	455 (17.06)
	It’s complicated	161 (6.04)
**Device used**		
	Mobile phone	2266 (84.96)
	Tablet	54 (2.02)
	Computer	347 (13.01)

#### Evaluation Study (Randomized Controlled Trial)

**Figure 3:**
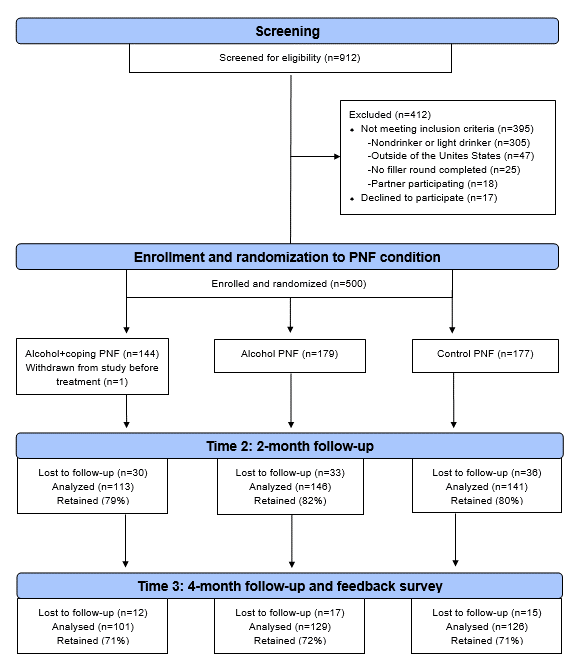
Flow of the evaluation showing study participants through screening, enrollment, and follow-ups. PNF: personalized normative feedback. T1: time point 1; T2: time point 2; T3: time point 3.

The third monthly round of LezParlay inquired about alcohol use and LBQ stigma exposure and served as the screening instrument and baseline assessment (time point [T1]) for the randomized controlled trial (RCT). As players completed this round, a subsample of 500 LBQ drinkers meeting the eligibility criteria were invited to take part in an evaluation study wherein they were incentivized to play subsequent rounds and complete a feedback survey following the competition. A total of 1337 LBQ women completed the round with 912 users covertly screened for eligibility based on their responses to round questions about alcohol use (ie, reporting drinking ≥3 days per week or consuming ≥3 drinks on their heaviest drinking occasion) and other app data (eg, geolocation in the United States, at least one previous round played, and no partner participating) before the 500 spots in the evaluation study were filled. As described in greater detail elsewhere [43], at the point of study enrollment, participants were covertly randomized to receive 1 of 3 sequences of PNF delivered at the end of the third and fourth monthly rounds: alcohol + coping, alcohol + control, or control topics only. Reductions in drinking and negative consequences were assessed 2 (time point 2 [T2], June 2019) and 4 (time point 3 [T3], August 2019) months later. Following completion of a postcompetition feedback survey, participants were debriefed regarding the research questions and the nonrandom nature of the topics on which they received detailed results in 2 of the 8 competition rounds. A CONSORT (Consolidated Standards of Reporting Trials) diagram summarizing the flow of participants through the RCT portion of the trial is presented in Figure 3 (see also Multimedia Appendix 1 for this trial’s CONSORT E-HEALTH checklist). Mirroring the larger user base, the evaluation study drinkers were diverse in terms of their geographic locations, representing 44 US states and 221 different counties and age groups, sexual identities, races, and ethnicities.

### Ethical Considerations

Human subjects approval for this research was granted by the Loyola Marymount University institutional review board (protocol #LMUIRB2018SU14) on August 14, 2018. All procedures [43] were in accordance with the ethical standards of the US Department of Health and Human Services Office for Human Research.

### Measures

#### Competition Engagement

Data collected by Google Analytics and the application examined the total number of users who signed up to participate in the LezParlay competition and detailed users’ average number of logins, number of rounds completed, and most visited areas of the app.

#### Demographics

All users reported their sexual identity and relationship status at competition sign-up. Age in years, race, and ethnicity were also reported by the evaluation study participants at the point of study enrollment.

#### Perceived Norms for Alcohol Use and Negative Consequences

Three items modeled after the Quantity, Frequency, Max measure [47] assessed perceived LBQ peer alcohol use norms for the average number of drinks consumed in a typical week (2 items), maximum (or peak) number of drinks consumed on one occasion (1 item), and the number of negative alcohol-related consequences experienced (1 item). The perceived norm for weekly drinks comprised 2 items that prompted users to report their perception of the number of days per week the typical user drank (0-7 days) and the typical user’s average number of drinks consumed per drinking day (0 to ≥12 drinks). The product of these 2 items was computed to create a variable indicative of users’ perceived norm for weekly drinks. For the peak drinking norm, users reported the typical user’s maximum number of drinks consumed on one occasion (0 to ≥12 drinks). To assess the perceived norm for negative consequences, users were presented with a list of 8 negative consequences (eg, had a hangover or illness, got in a physical or verbal fight, had problems with a significant other, missed a social engagement or event, had problems with friends or family, performed poorly at work or school, had problems with money, and had an unwanted or regrettable sexual experience) and were then asked to report the number they thought the typical user experienced due to drinking or partying. At all 3 time points, items assessing norms referenced the previous 2 months, and the sexual identity and age group of the typical user in these questions were piped to match each user’s own sexual identity and age group (eg, “Over the past two months, on average, on how many days per week did the typical [lesbian] user in her [30s] drink?”).

#### Own Alcohol Use and Negative Consequences

Users’ own weekly drinks, peak drinks on one occasion, and negative consequences in the past 2 months were assessed using items that paralleled norm items (eg, “Over the past two months, on average, on how many days per week did YOU drink?”) at the same time points and presented the same response options (ie, 0-7 days; 0 to ≥12 drinks; 0-8 negative consequences).

#### Interpersonal Stigma Exposure

Interpersonal stigma exposure was assessed at T1 with two items adapted for the in-game context from a widely used measure of sexual minority stigma [48]: (1) “During the past 2 months, how many times have you been physically harmed due to your sexual identity?” (2) “During the past 2 months, how many times have you been verbally harassed or threatened (online or in person) due to your sexual identity?” More than 75% of users’ responses were concentrated in the range of 0 to 1. Therefore, items were first recoded to reflect this binary (ie, 0=this did not happen; 1=this happened once or more times) and then summed to produce a score between 0 and 2. This measure was included as a covariate in statistical models evaluating efficacy due to sexual minority status–related violence and harassment being the experiences most consistently linked to alcohol consumption and negative alcohol-related consequences among sexual minority women [13-15].

#### LezParlay Acceptability

The postcompetition feedback survey prompted study participants to rate numerous aspects of the competition (the stereotype challenge concept, topics and questions, detailed results, leaderboards, the ability to browse player profiles, the ability to submit questions, the ability to bet points on the accuracy of guesses, SMS text messages, and email communications) on Likert-type scales ranging from *did not like at all* (rating=0) to *liked very much* (rating=5). Total acceptability scores were computed by summing the ratings.

#### LezParlay Perceived Benefits

A single item in the feedback survey asked evaluation study participants to select yes or no in response to the question, “Did you find taking part in LezParlay to be psychologically beneficial?” Those selecting *yes* in response were invited to enter text describing their perceived benefits.

#### LezParlay Ideas for Improvement

A final free-response question asked evaluation study participants to share any ideas they had for ways in which LezParlay could be improved (ie, “Do you have any ideas for how LezParlay could be improved? What would you like to see in the next version?”).

### Analysis Plan

#### Evaluating Feasibility

Descriptive statistics examined the level of engagement with the LezParlay app (ie, total number of sign-ups and average number of rounds played), initial levels of alcohol use among players, competition-derived actual norms for treatment topics, competition acceptability among drinkers taking part in the RCT, and the proportion of drinkers taking part in the RCT reporting perceived psychological benefits. Qualitative responses to items assessing the perceived benefits associated with LezParlay and ideas for improvement were examined using a generic inductive qualitative coding approach [49,50]. This iterative approach is similar to grounded theory but is more commonly used in the program evaluation literature, where the coding objective tends to be summarizing phenomena for basic understanding rather than building theory. As this approach can be prone to reflecting the biases of a single coder, multiple coder perspectives were sought, with a lesbian-identified senior researcher (SCB), a gay male–identified senior researcher (JWL), and 2 heterosexual female research assistants taking part in the coding process. For psychological benefits, coding sought to condense the raw text descriptions submitted by participants into a summary of common benefit categories. First, the 2 senior researchers (SCB and JWL) conducted independent, initial readings of participant responses, with each aiming to identify no more than 10 unique categories of benefits. As responses were generally short, although many described >1 benefit, it was decided a priori that each response could receive up to 3 category classifications. The senior researchers then met, compared categories, agreed upon common category themes, and identified several pairs of categories that were extremely similar and could be condensed into a single category. This process culminated in 6 shared benefit categories. Next, 2 research assistants independently classified all responses according to the 6 categories, with each response coded for a maximum of 3 benefits. Interrater reliability was high (κ=0.91), and discrepancies were resolved through discussion. A similar approach was used to code participants’ ideas for improving LezParlay. However, as the research team only sought to identify the most agreed-upon or frequently submitted ideas for improvement to inform the next version of the app, all 4 coders independently grouped participant responses in terms of similarity. No discrepancies in groupings were observed between coders, and similar responses were tallied for summary purposes.

#### Evaluating Efficacy

Preliminary analysis of RCT data examined the distributions of alcohol-related variables, the nature of missing data, and the characteristics of participants lost to follow-up (*t* tests). One-way ANOVAs and chi-square tests also established conditional equivalency for demographic characteristics, interpersonal stigma, perceived alcohol use norms, and alcohol use behaviors assessed at T1. Examination of attrition suggested that missingness was random rather than systematic. As such, 3 multilevel models, each with maximum likelihood estimation to deal with data missing at random, a random intercept component, and an unstructured covariance matrix, were conducted in SAS (version 9.4) to assess the effects of treatment PNF (alcohol PNF and alcohol + coping PNF) on respective changes in weekly drinks, peak drinks, and negative consequences relative to control PNF. In all 3 models, predictors included study condition (alcohol PNF and alcohol + coping PNF vs control PNF) and time (T2 and T3 vs T1). To determine whether there were changes in drinking outcomes over time related to PNF treatment, condition × time interaction terms were included in each model. Covariates also included in the models were age, sexual identity (bisexual and queer vs lesbian), race (White vs non-White), ethnicity (non-Hispanic or Latino vs Hispanic or Latino), relationship status (single vs in a relationship or married), and exposure to interpersonal LBQ stigma. Post hoc Tukey tests were then conducted to determine the nature of significant condition × time effects.

## Results

### Research Question 1: Were LBQ Women Engaged by the LezParlay Competition?

Yes, despite no traditional study incentives being offered at recruitment, sign-up, or initial round completion, 2667 LBQ women signed up and played ≥1 round. Furthermore, the average user logged into the LezParlay app 2 times during the competition following initial sign-up; completed 1.97 rounds; and, on average, spent 4.15 minutes on the app per login (no SDs available). The LezParlay web application also recorded 54,072 total page visits among logged in users, with the most frequented sections of the app devoted to browsing the social media–inspired profiles of other users, followed by playing monthly rounds, viewing detailed results (ie, PNF), and viewing leaderboards.

### Research Question 2: Was It Feasible to Derive Risk-Reducing LBQ Actual Drinking Norms From In-Round Questions?

Yes, of the 1337 LezParlay users who completed the round where alcohol use was first assessed, 254 (19%) reported no alcohol consumption or light drinking (≤2 drinks per week), and 346 (25.88%) reported moderate drinking (3-7 drinks per week and ≤3 drinks on any day). Notably, ≤7 drinks per week and ≤3 drinks per day are the upper limits for low-risk drinking among women, as defined by the National Institute on Alcohol Abuse and Alcoholism [51], as these patterns of consumption equate to low risks for alcohol dependence and development of alcohol-related health problems. Higher levels of risk were also well represented in LezParlay, with 55.12% (737/1337) of the users who completed this round consuming ≥8 drinks per week or ≥4 drinks on any day, thereby meeting the National Institute on Alcohol Abuse and Alcoholism’s definition of high-risk drinking [51]. Conferring elevated risks for AUD and alcohol-related health problems, the average number of drinks consumed per week among these users ranged from 8 to 56 drinks, and peak drinks consumed on a day ranged from 4 to ≥12 drinks for the 2-month period referenced in the game questions. However, as LezParlay users were so diverse in their patterns of alcohol consumption, the broader composition of alcohol use among users was sufficient for generating risk-reducing actual norms to deliver to drinkers in the evaluation study. As is typically the case in traditional PNF interventions, the lower levels of consumption among nondrinkers and low-risk drinkers attenuated the higher levels of consumption among high-risk users. Round-derived actual norms featured in treatment PNF are presented in Table 2.

**Table 2:** Competition-derived actual norms presented in treatment personalized normative feedback.

Norms			Age groups (years)				
			21-29		30-39		≥40
**Round 3: alcohol use actual norms^a^**			n=627		n=498		n=212
	Drinking days per week, mean	2		2		1.5	
	Drinks per occasion, mean	2.5		2		2	
	Weekly drinks, mean	5		4		3	
	Peak drinks on one occasion, mean	4		3		3	
	Negative consequences, mean	2		1.5		1	
**Round 4: coping actual norms^a^**			n=503		n=414		n=186
	Times drank alcohol to cope, %	18		17		16	
	Time used drugs to cope, %	12		9		9	
	Times exercised or meditated to cope, %	55		61		49	
	Times sought social support to cope, %	53		50		62	

^a^As no sexual identity differences were observed for alcohol use or coping behaviors, participants received the same age group–specific lesbian, bisexual, and queer actual norms for these topics as a function of condition assignment.

### Did PNF on Alcohol Use Delivered Within the Competition Reduce Alcohol-Related Risks Among LBQ Drinkers? Was It More Beneficial to Deliver PNF on Both Drinking and Coping Behaviors Than on Drinking Behaviors Alone?

Retention in the RCT was adequate, with 80.2% (400/499) of the evaluation study participants retained at T2 and 71.3% (356/499) at T3. Participants lost to follow-up were younger (t_497_=4.48; *P*<.001) and non-Hispanic White (t_497_=4.13; *P*<.001). Attrition, in this case, was considered random rather than systematic, given that the *average* participant in the study was both younger and non-Hispanic White, and attrition was not significantly associated with any other study variables. Beyond attrition, there were no other missing data among participants. As shown in Table 3, tests of conditional equivalency revealed no significant between-condition differences for any of the variables at baseline.

The results for multilevel models, predicting weekly drinks, peak drinks on one occasion, and negative alcohol-related consequences are presented in Table 4. The condition × time effects in each model were significant, indicating that treatment PNF conditions predicted significant changes in outcomes over time, controlling for baseline covariates (ie, sexual identity, race, ethnicity, age, relationship status, and interpersonal stigma exposure).

**Figure 4:**
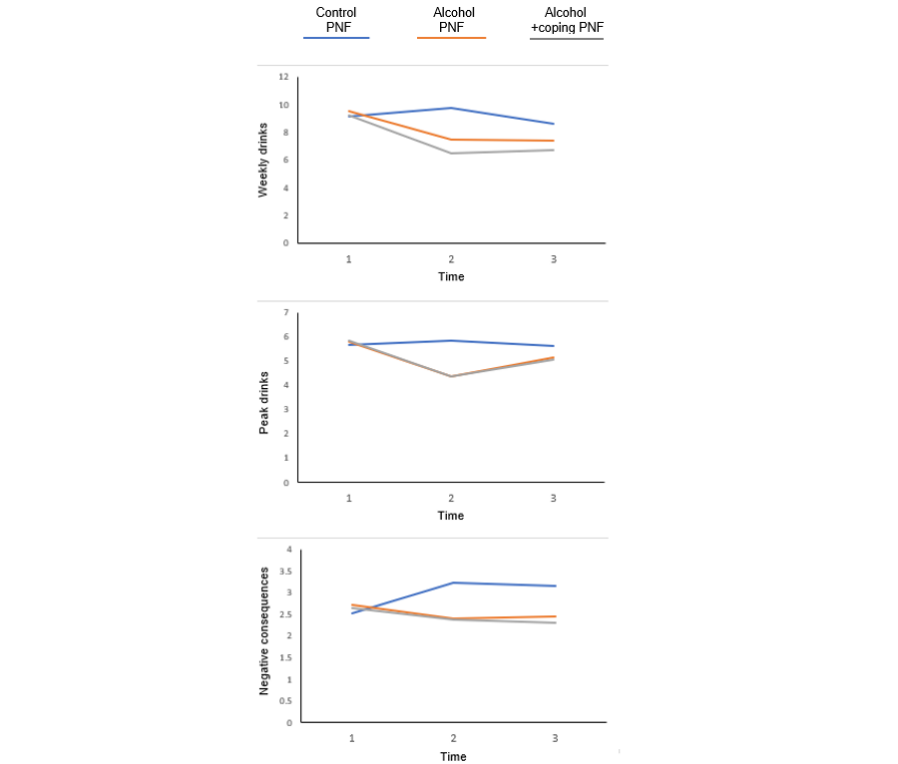
Personalized normative feedback (PNF) condition as a function of time for each outcome.

As presented in Table 5 and Figure 4, post hoc analyses probing interaction effects for each outcome revealed that participants in both treatment PNF conditions significantly decreased their weekly drinks from T1 to T2 relative to participants receiving PNF on control topics, but significant differences in weekly drinks were retained only at T3 between the alcohol + coping PNF and control PNF conditions. Similarly, both treatment PNF conditions predicted significant decreases in peak drinks consumed from T1 to T2 relative to control PNF; however, only the differences between the alcohol + coping and control PNF conditions met the threshold for significance at T3 (*P*=.06 for alcohol PNF vs control PNF). Finally, participants in both treatment PNF conditions significantly decreased the negative consequences they experienced relative to controls from T1 to T2 and from T2 to T3.

**Table 3:** Baseline demographics, psychosocial characteristics, drinking norms, and alcohol use of evaluation study participants overall and by condition assignment.

Characteristics			Overall (N=499)		Control PNF^a^ (n=177)		Alcohol PNF (n=179)		Alcohol + coping PNF (n=143)
**Sexual identity, n (%)**									
	Lesbian	290 (58.1)		94 (53.1)		108 (60.3)		89 (62.2)	
	Bisexual	115 (23)		48 (27.1)		39 (21.8)		29 (20.2)	
	Queer	94 (18.8)		35 (19.8)		32 (17.9)		25 (17.5)	
**Relationship status, n (%)**									
	Single	209 (41.9)		80 (45.2)		69 (38.5)		60 (41.9)	
**Ethnicity, n (%)**									
	Hispanic/Latino	123 (24.6)		40 (22.6)		46 (25.7)		37 (25.8)	
**Race, n (%)**									
	American Indian/Alaskan Native	13 (2.6)		4 (2.3)		5 (2.8)		4 (2.7)	
	Asian American	39 (7.8)		17 (9.6)		16 (8.9)		6 (4.1)	
	Black/African American	70 (14)		26 (14.7)		25 (14)		19 (13.2)	
	Hawaiian/Pacific Islander	1 (0.2)		0 (0)		0 (0)		1 (0.6)	
	White	268 (53.7)		99 (55.9)		91 (50.8)		78 (54.5)	
	Multiracial	53 (10.6)		15 (8.5)		23 (12.8)		15 (10.4)	
	Other	55 (11)		16 (9)		19 (10.6)		14 (20)	
Age (years), mean (SD)			29.87 (7.32)		29.47 (7.03)		30.37 (7.75)		29.73 (7.15)
T1^b^ interpersonal stigma, mean (SD)			0.61 (0.69)		0.66 (0.69)		0.56 (0.66)		0.62 (0.70)
**T1 perceived drinking norms, mean (SD)**									
	Norm–weekly drinks	13.94 (9.37)		13.84 (9.44)		14.07 (10.35)		13.89 (7.92)	
	Norm–peak drinks	6.31 (2.18)		6.16 (2.27)		6.36 (2.07)		6.43 (2.20)	
	Norm–consequences	2.88 (1.74)		2.84 (1.65)		3.01 (1.84)		2.73 (1.72)	
**T1 alcohol use, mean (SD)**									
	Weekly drinks	9.15 (7.51)		9.13 (7.90)		8.96 (8.19)		9.43 (6.00)	
	Peak drinks	5.79 (2.34)		5.74 (2.43)		5.76 (2.37)		5.87 (2.19)	
	Consequences	2.52 (1.89)		2.45 (1.95)		2.55 (1.86)		2.58 (1.89)	

^a^PNF: personalized normative feedback.

^b^T1: time point 1.

**Table 4:** Multilevel model results for outcomes (weekly drinks, peak drinks, and negative alcohol-related consequences).

Outcomes	Weekly drinks			Peak drinks			Consequences	
	b (SE)	*P* value	b (SE)		*P* value	b (SE)		*P* value
Alcohol PNF^a^	0.41 (0.74)	.58	0.13 (0.22)		.54	0.2 (0.16)		.24
Alcohol + coping PNF (reference: control PNF)	0.14 (0.78)	.86	0.16 (0.23)		.49	0.13 (0.17)		.47
Time 2	0.65 (0.39)	.09	0.19 (0.14)		.19	0.71 (0.12)		<.001
Time 3 (reference: time 1)	−0.50 (0.40)	.22	−0.05 (0.15)		.73	0.64 (0.13)		<.001
Alcohol PNF × time 2	−2.72 (0.54)	<.001	−1.61 (0.20)		<.001	−1.03 (0.18)		<.001
Alcohol PNF × time 3	−1.64 (0.57)	.004	−0.59 (0.21)		.005	−0.90 (0.18)		<.001
Alcohol + coping PNF × time 2	−3.39 (0.58)	<.001	−1.67 (0.22)		<.001	−1.00 (0.19)		<.001
Alcohol + coping PNF × time 3	−2.03 (0.61)	.01	−0.71 (0.23)		.02	−0.98 (0.20)		<.001
Queer	−1.88 (0.78)	.02	−0.29 (0.22)		.19	−0.48 (0.16)		.01
Bisexual (reference: lesbian)	−1.26 (0.72)	.08	0.40 (0.20)		.05	−0.03 (0.15)		.83
Non-White (reference: White)	0.75 (0.65)	.25	0.23 (0.18)		.21	0.51 (0.13)		.001
Hispanic or Latinx (reference: non-Hispanic or Latinx)	−0.68 (0.77)	.38	0.02 (0.22)		.93	−0.28 (0.15)		.07
Age	−0.05 (0.04)	.20	−0.04 (0.01)		.001	−0.04 (0.01)		<.001
Single (reference: coupled or married)	2.30 (0.62)	.002	0.61 (0.18)		.01	0.41 (0.13)		<.001
Interpersonal stigma exposure	1.85 (0.43)	<.001	0.38 (0.12)		.002	0.45 (0.09)		<.001

^a^PNF: personalized normative feedback.

**Table 5:** Tukey post hoc test results probing PNF^a^ condition × time interactions.

PNF condition comparisons		Weekly drinks			Peak drinks			Consequences	
		b (SE)	*P* value	b (SE)		*P* value	b (SE)		*P* value
**T1^b^**									
	Alcohol vs control	−0.41 (0.74)	.58	−0.13 (0.22)		.54	−0.20 (0.16)		.24
	Alcohol + coping vs control	−0.14 (0.78)	.86	−0.16 (0.23)		.49	−0.13 (0.17)		.47
	Alcohol vs alcohol + coping	0.27 (0.78)	.73	−0.03 (0.23)		.91	0.07 (0.17)		.69
**T2^c^**									
	Alcohol vs control^d^	2.31 (0.77)	.003	1.48 (0.23)		<.001	0.83 (0.18)		<.001
	Alcohol + coping vs control^d^	3.25 (0.82)	<.001	1.51 (0.25)		<.001	0.86 (0.19)		<.001
	Alcohol vs alcohol + coping	0.94 (0.82)	.25	0.04 (0.25)		.88	0.03 (0.19)		.87
**T3^e^**									
	Alcohol vs control^d^	1.24 (0.79)	.12	0.46 (0.24)		.06	0.70 (0.19)		.002
	Alcohol + coping vs control^d^	1.90 (0.84)	.03	0.55 (0.26)		.03	0.86 (0.20)		<.001
	Alcohol vs alcohol + coping	0.66 (0.84)	.43	0.10 (0.26)		.71	0.15 (0.20)		.44

^a^PNF: personalized normative feedback.

^b^T1: time point 1.

^c^T2: time point 2.

^d^Across outcomes, Cohen *d* effect size estimates for significant treatment versus control comparisons ranged from 0.20 to 0.33 at T2 and 0.12 to 0.22 at T3.

^e^T3: time point 3.

### Did LBQ Drinkers Find the Competition Acceptable and Psychologically Beneficial? What Ideas for Improvements Did They Submit?

Overall, drinkers in the evaluation study found the LezParlay competition to be highly acceptable, with the average participant rating competition aspects between *liked* and *liked very much* (mean 41.26, SD 3.84; out of a maximum score of 50). Table 6 presents descriptive statistics for individual acceptability items. Notably, the highest-rated aspect was *receiving detailed results each round* (mean 4.51, SD 0.56). The exploratory 1-way ANOVA and correlational analyses also determined that acceptability ratings were not significantly associated with participants’ study condition (*F*_2,355_=0.41, *P*=.67) or baseline measures of alcohol outcomes (*r* ranged from 0.02 to 0.04; *P* ranged from .64 to .68).

Of the 356 participants who completed the feedback survey, 331 (93%) reported finding the LezParlay competition to be psychologically beneficial. Furthermore, 85.5% (283/331) of the participants that indicated benefits entered text to describe the experienced benefits. Qualitative coding resulted in 6 common categories of benefits reported by participants: knowledge and social comparison, community connection and identity strength, stigma reduction, introspection and self-confrontation, entertainment and fun, and mood enhancement. Table 7 presents the proportion of total responses that reflected each benefit category and representative responses of benefits in each category.

Notably, although benefits associated with social comparison and self-confrontation in the domain of drinking may be experienced in a traditional PNF alcohol intervention, benefits associated with community connection and identity strength, stigma reduction, entertainment, and enhanced mood and outlook would not be experienced in the context of traditional PNF. Presumably, these extra psychological benefits described by participants were related to LezParlay’s social media–inspired web-based community features, the broad challenging of negative LBQ stereotypes via PNF, and the competition-fostering constellation of game mechanics.

In response to the optional item inquiring as to how LezParlay could be improved, 47.5% (169/356) of participants submitted a total of 307 individual ideas for improvement. The most frequently submitted ideas revealed that participants most commonly desired a native (iOS and Android) smartphone app for the competition (112/307, 36.5%); more frequent rounds (eg, weekly rather than monthly) with faster results delivery (74/307, 24.1%); increased opportunity for interaction between players (eg, a chat feature or direct messaging that could be turned on and off; 43/307, 14%); increased ease of inviting friends and the ability to earn bonus points for referring friends (24/307, 7.8%); the ability to go back and change previously submitted guesses or point wagers before a round closing (12/307, 3.9%); the connection of results to informational articles or community resources (11/307, 3.6%); additional questions about race, gender identity, and sexual identity–based biases within the community (9/307, 2.9%); worldwide promotion and additional results comparing the behaviors and experiences of LBQ players in different countries or regions (4/307, 1.3%); and the ability to see the community thumbnail photo collage of LBQ users being guessed about on the *guess question* screen rather than on a previous screen (4/307, 1.3%).

**Table 6:** LezParlay competition acceptability ratings by item (N=356).

Acceptability item	Rating, mean (SD)^a^
The “stereotype challenge” concept	4.37 (0.56)
The topics and questions	4.01 (0.56)
Receiving the detailed results each round	4.51 (0.56)
Browsing players profiles	3.62 (0.66)
Submitting and voting on questions	3.95 (0.59)
Betting points on your guesses being correct	4.34 (0.62)
Receiving SMS text message reminders	4.12 (0.58)
Receiving email reminders	4.27 (0.50)
Viewing the top scorer leaderboards	3.89 (0.60)
Competing for money and receiving gift cards	4.09 (0.55)

^a^Response options ranged from 1=disliked very much to 5=liked very much.

**Table 7:** Categories of psychological benefits described by participants and representative responses (n=283).

Benefit category	Total, n (%)	Representative responses and user characteristics
Knowledge and social comparison	184 (65)	“I work at an LGBTQ community center and it really helped having data to influence our programs and identify topics/issues to discuss in our women's group meetings.” [Queer, 41 years] “Let me learn more about the lgbtq community and see that I drink way more than average lol fail” [Lesbian, 38 years]
Community connection and identity strength	96 (33.9)	“Being in my 50s and feeling sort of invisible these days this competition really helped me feel connected to something again” [Lesbian, 52 years] “It was so great for me although it's hard to describe how/why exactly...felt connected and in the know...also felt more confident and secure in my identity.” [Queer, 25 years]
Stigma reduction	88 (31)	“This really helped me reduce biases that I had internalized without even realizing it!” [Queer, 26 years] “Cool to see that some of the negative ways we get portrayed in the media are totally off.” [Lesbian, 36 years]
Introspection and self-confrontation	66 (23.3)	“This really helped me see that I need to get my shit together in several areas” [Lesbian, 33 years] “Made me question some of my own tendencies and behaviors. Came to see that I was doing what I thought everyone else was doing which wasn't even the truth...” [Lesbian, 28 years]
Funa and entertainment	54 (19)	“Loved the competition, betting, prizes, and leaderboards... so so fun” [Lesbian, 51 years] “It was so fun and I was able to get my lesbian roommate to play with me...we got super competitive about scores and had a blast!” [Bisexual, 37 years]
Mood and outlook enhancement	40 (14.1)	“...helped my mental health and gave me a more positive outlook on all things queer.” [Queer, 29 years] “It was a source of enjoyment. Getting the results always put me in such a good mood...even when I was wrong about stuff...” [Lesbian, 23 years]

## Discussion

### Principal Findings

LezParlay leveraged gamification and deep cultural adaptations to deliver a PNF alcohol intervention to LBQ women, a difficult-to-engage population for whom alcohol-related risks are high, but efficacious evidence-based intervention and prevention programs are lacking [3,52]. Reflecting the widespread appeal and cost-efficacy afforded by LezParlay’s framing as a competition designed to challenge negative LBQ stereotypes, a very large and diverse group of LBQ women signed up to take part in the competition despite the lack of traditional study incentives being offered for sign-up or initial round play. Furthermore, more than half of the LBQ users who completed the round in which alcohol use was first assessed exceeded established drinking guidelines for women and thus were an ideal population for PNF intervention. LBQ women taking part in LezParlay substantially overestimated LBQ-specific peer norms for drinking, experiencing negative consequences, and engaging in maladaptive coping behaviors in response to stress and stigma consistent with previous survey study findings [16-19]. In summary, the markedly lower levels of consumption among alcohol abstainers and low-risk drinkers also taking part in the round attenuated the levels of consumption among heavier drinkers, allowing risk-reducing actual drinking norms (presented in PNF) to be organically generated in real time from users’ round data.

This novel approach to PNF intervention also demonstrated efficacy in reducing drinking and its negative consequences. Relative to LBQ drinkers randomized to receive PNF on control topics in the competition, those who received treatment PNF on drinking or both drinking and coping similarly and substantially reduced their weekly drinks, peak drinks consumed on one occasion, and number of negative consequences 2 months later. For these outcomes, effect sizes associated with LezParlay treatment arms at the 2-month follow-up were consistent with or exceeded the short-term effects associated with treatment arms of traditional, remotely delivered PNF alcohol interventions in other populations [53-55]. Thus, in the short term, the impact of additional treatment PNF on coping behaviors beyond alcohol PNF was negligible. However, at the 4-month follow-up, relative to control PNF, the reductions in quantity of consumption outcomes (ie, weekly drinks and peak drinks) associated with the alcohol-only PNF condition faded, whereas they remained relatively robust in the alcohol + coping condition. There are 2 potential explanations for this finding. First, as previous research has found drinking to cope to be a strong overall predictor of alcohol consumption among LBQ women [56,57], correcting LBQ coping norms may have changed participants’ own coping behaviors to be more adaptive, which, with passing time, impacted alcohol use outcomes. However, given the design of this trial, wherein coping PNF corrected norms for coping-motivated drinking (among other behaviors) and was delivered 1 month following the initial treatment PNF on alcohol use, it is possible that coping PNF had little effect on participants’ subsequent coping behaviors. Rather, the portion of coping PNF that corrected norms for coping-motivated drinking may have acted as a broader *booster* to alcohol PNF, further reinforcing the idea that LBQ peers do not drink as much as one previously thought. Thus, although efficacy findings from this initial trial are promising and suggest that both alcohol and coping PNF are beneficial, additional research will be needed to fully understand the mechanisms (ie, correcting coping norms or reinforcing actual alcohol use norms) by which coping PNF influences drinking in this population.

In addition to being a feasible and effective means of delivering PNF to this population, LBQ drinkers also found the LezParlay competition to be both highly acceptable and psychologically beneficial. Notably, the detailed results (ie, PNF) were the most liked aspect of LezParlay, and ratings were not significantly associated with study condition or baseline alcohol consumption. Thus, those receiving fewer and more health-related results as a function of condition and those entering the study as lighter and heavier drinkers similarly enjoyed receiving the PNF delivered. These findings suggest that future versions of the competition might also correct additional types of alcohol and coping-related norms or expand the topics on which PNF is delivered to other areas of physical and mental health without detracting from acceptability or engagement. Most participants also reported that they psychologically benefited from taking part in the competition, and descriptions of benefits reflected learning and social comparison, community connection and identity strength, stigma reduction, enhanced mood or outlook, and entertainment. Many of these benefits map onto LezParlay’s social media–inspired web-based community features, the constellation of game mechanics, and the broad challenging of negative LBQ stereotypes and, importantly, extend far beyond the psychological benefits associated with traditional PNF alcohol interventions. Finally, participants submitted several actionable ideas for ways in which the LezParlay app, user experience, and competition format could be improved. These insights will inform the next version of LezParlay.

### Implications for Intervention Research and Practice

To date, alcohol interventions developed for sexual minorities have tended to be clinical, intensive, and focused on affirming sexual identities, aiding individuals in understanding sexual minority stress processes, and providing resources to help individuals cope with stigma more adaptively [52,58,59]. Although these approaches hold much promise for individuals seeking treatment, other findings suggest that the central and highly visible positions that bars and nightclubs occupy in sexual minority communities may diminish community members’ recognition of their heavy drinking as problematic and motivation to change, thereby deterring or delaying treatment seeking [60-62]. However, very few, if any, previous evidence-based interventions have been designed to motivate reductions in drinking among sexual minorities who do not view their drinking as problematic or experience other barriers to intensive treatment programs. To our knowledge, this study is the first to demonstrate that correcting sexual minority–specific drinking and coping norms via PNF is effective in reducing drinking in a sexual minority population. Although more research is needed, these findings suggest that the impact of PNF is not diminished by violence and harassment due to sexual minority status and that this approach may similarly reduce alcohol-related risks in other populations of sexual minority adolescents and adults. Importantly, for LBQ women, this gamified, incognito, brief intervention also provides a valuable complement to more intensive programs being developed to meet the needs of self-aware LBQ women already motivated to reduce their consumption [7] and those seeking culturally tailored treatment for AUD and comorbid mental health problems [8].

The present findings also bring clarity to confusion in the substance use literature around the appropriateness and utility of social norms interventions for health disparity populations [18]. For instance, because it is well-known that LBQ women disproportionately drink and experience consequences relative to heterosexual women, there is often confusion as to whether delivering PNF on LBQ-specific actual alcohol use norms would have the effect of increasing or reducing drinking. As evident from this trial’s findings, increasing drinking should not be a concern with this approach to the extent that LBQ women overestimate the drinking of other LBQ women; that is, similar to college students known to drink disproportionately drink relative to noncollege students and military populations known to drink more than their civilian peers, PNF reduces alcohol use in these heavy drinking populations despite disparate out-group comparisons. This type of intervention is effective because perceived in-group drinking norms are both highly relevant to the self and substantially overestimated.

This study’s feasibility findings also provide an innovative answer to challenges related to reaching and engaging stigmatized minority populations with PNF in the real world. LezParlay delivered the core components of a PNF intervention within a fun, culturally tailored digital competition designed to challenge negative stereotypes about the target population. This gamified, incognito intervention approach was found to be highly engaging, acceptable, and psychologically beneficial among alcohol-consuming LBQ women and meaningfully reduced their alcohol-related risks. Although more research is needed, the stereotype challenge concept, along with the injection of established game mechanics and cultural themes, may have similar utility in reaching other high-risk stigmatized minority groups with PNF on drinking and other health risk behaviors. Finally, looking past PNF, findings from this study also suggest that challenging negative identity-related stereotypes and including web-based community features may also prove fruitful in minority, stress-informed digital health and mental health programs targeting internalized stigma, loneliness, and isolation [8,63,64].

### Limitations and Future Directions

As the initial step in a new direction for alcohol intervention development, the key limitations associated with this study include the relatively short duration of the follow-up period, organic assessment of baseline and follow-up alcohol outcomes within rounds of the competition at T1 and T2, and assessment of acceptability and psychological benefits only among LBQ drinkers involved in the RCT. Thus, future evaluation efforts should follow participants for a longer duration (6-24 months), incorporate survey-based baseline and follow-up assessments, and examine the acceptability and psychological benefits among nondrinkers and other LBQ players not involved in the efficacy portion of the trial. An additional limitation to be remedied in future research is this trial’s lack of an assessment-only control condition. Although randomizing participants to receive PNF on either treatment or control topics, as was done in this study, reflects the gold standard trial design in the PNF intervention literature, it may not be optimal when PNF is delivered within a culturally tailored digital competition focused on challenging negative group stereotypes. That is, participants in all 3 PNF conditions described unanticipated, far-reaching psychological benefits associated with broader participation in the competition, including stigma reduction, community connection, and identity strength. As these factors are theorized to diminish the degree to which sexual minority stress negatively impacts health risk behavior [65,66], it is possible that they alone may have reduced drinking across PNF conditions to some degree. To fully determine the impact of the LezParlay competition app as an alcohol intervention strategy, it will be important for future trials to also include an assessment-only control group with no exposure to PNF or the competition app. Future trials using such an expanded design should examine internalized stigma, LBQ identity strength, and community connection, in addition to perceived norms for treatment topics as potential mediators of conditional effects on drinking and negative consequences. Similarly, it will also be important to examine internalized, structural, and interpersonal forms of sexual minority stigma as potential moderators of direct and indirect effects.

Although this efficacious initial version of LezParlay was a standalone intervention focused exclusively on correcting descriptive drinking and coping norms, exciting directions for future research also lie in the prospect of incorporating additional components to further reduce alcohol-related risks and increase wellness more broadly. For example, future research may seek to evaluate the utility of including a judgment-based reflective injunctive alcohol normative feedback component that builds on promising pilot findings among college students [41]. The competition’s multiround format also provides a natural environment for examining the utility of PNF on dynamic or *trending* health-related norms [67,68] focused on group-based changes in behavior or attitudes over time. Furthermore, the competition’s ability to attract and engage LBQ women in the absence of traditional study incentives also suggests that it could play a future role in implementing more intensive health interventions that have found it difficult to engage this population [36]. Thus, another important direction for future research is to examine the degree to which LezParlay could fruitfully serve to attract LBQ women and motivate behavior change as part of a larger multicomponent intervention targeting multiple health behaviors. For instance, within the competition, PNF could target additional health behaviors, and after motivating behavior change through norms correction, the app could link at-risk users to intensive web-based intervention components or local health promotion programs that correspond to these behaviors.

### Conclusions

The results of this hybrid trial provide initial support for the feasibility and efficacy of LezParlay as a culturally tailored, gamified, PNF alcohol intervention for LBQ women, thereby narrowing costly disparities in alcohol intervention research and practice. The reductions in alcohol use and negative consequences associated with PNF on drinking and coping delivered within LezParlay demonstrate the utility of PNF as an alcohol intervention strategy for a stigmatized minority health disparity population. These findings should behoove substance use researchers developing interventions for sexual minorities to consider such sexual identity–specific peer norms as potential intervention targets. Furthermore, to overcome engagement challenges associated with delivering PNF to non–treatment-seeking members of stigmatized minority groups and broaden the psychological benefits associated with this strategy, the findings underscore the value of packaging PNF within a broader culturally tailored competition designed to challenge negative group stereotypes.

## Appendix

Multimedia Appendix 1 CONSORT-eHEALTH checklist (V 1.6.1).

## Abbreviations

AUD: alcohol use disorder
CONSORT: Consolidated Standards of Reporting Trials
LBQ: lesbian, bisexual, and queer
PNF: personalized normative feedback
RCT: randomized controlled trial
T1: time point 1
T2: time point 2
T3: time point 3
