# Supplementary material for: A Gamified Personalized Normative Feedback App to Reduce Drinking Among Sexual Minority Women: Randomized Controlled Trial and Feasibility Study
Source: J Med Internet Res. 2022 May 13;24(5):e34853. doi: 10.2196/34853 (PMC9143770; doi:10.2196/34853)
Supplement: Multimedia Appendix 1 [file jmir_v24i5e34853_app1.pdf]

CONSORT-EHEALTH (V 1.6.1) - Submission/Publication Form

The CONSORT-EHEALTH checklist is intended for authors of randomized trials evaluating web-based and Internet-based applications/interventions, including mobile interventions, electronic games (incl multiplayer games), social media, certain telehealth applications, and other interactive and/or networked electronic applications. Some of the items (e.g. all subitems under item 5 - description of the intervention) may also be applicable for other study designs.

The goal of the CONSORT EHEALTH checklist and guideline is to be
a) a guide for reporting for authors of RCTs,
b) to form a basis for appraisal of an ehealth trial (in terms of validity)

CONSORT-EHEALTH items/subitems are MANDATORY reporting items for studies published in the Journal of Medical Internet Research and other journals / scientific societies endorsing the checklist.

Items numbered 1., 2., 3., 4a., 4b etc are original CONSORT or CONSORT-NPT (non-pharmacologic treatment) items.
Items with Roman numerals (i., ii, iii, iv etc.) are CONSORT-EHEALTH extensions/clarifications.

As the CONSORT-EHEALTH checklist is still considered in a formative stage, we would ask that you also RATE ON A SCALE OF 1-5 how important/useful you feel each item is FOR THE PURPOSE OF THE CHECKLIST and reporting guideline (optional).

Mandatory reporting items are marked with a red *.
In the textboxes, either copy & paste the relevant sections from your manuscript into this form - please include any quotes from your manuscript in QUOTATION MARKS,
or answer directly by providing additional information not in the manuscript, or elaborating on why the item was not relevant for this study.

YOUR ANSWERS WILL BE PUBLISHED AS A SUPPLEMENTARY FILE TO YOUR PUBLICATION IN JMIR AND ARE CONSIDERED PART OF YOUR PUBLICATION (IF ACCEPTED).
Please fill in these questions diligently. Information will not be copyedited, so please use proper spelling and grammar, use correct capitalization, and avoid abbreviations.

DO NOT FORGET TO SAVE AS PDF _AND_ CLICK THE SUBMIT BUTTON SO YOUR ANSWERS ARE IN OUR DATABASE !!!

Citation Suggestion (if you append the pdf as Appendix we suggest to cite this paper in the [illegible]

Your response is too large. Try shortening some answers.

CONSORT-EHEALTH: Improving and Standardizing Evaluation Reports of Web-based and

Mobile Health Interventions
J Med Internet Res 2011;13(4):e126
URL: http://www.jmir.org/2011/4/e126/
doi: 10.2196/jmir.1923
PMID: 22209829

* Required

Your name *

First Last

Sarah Boyle

Primary Affiliation (short), City, Country *

University of Toronto, Toronto, Canada

Loyola Marymount University, Los Angeles, Cal

Your e-mail address *

abc@gmail.com

sarah.boyle@lmu.edu

Title of your manuscript *

Provide the (draft) title of your manuscript.

A Gamified, Social Media Inspired, Web-based Personalized Normative Feedback Alcohol Intervention for Lesbian, Bisexual, and Queer Women: Protocol for a Hybrid Trial.

Your response is too large. Try shortening some answers.

Name of your App/Software/Intervention *

If there is a short and a long/alternate name, write the short name first and add the long name in brackets.

LezParlay

Evaluated Version (if any)

e.g. "V1", "Release 2017-03-01", "Version 2.0.27913"

Your answer

Language(s) *

What language is the intervention/app in? If multiple languages are available, separate by comma (e.g. "English, French")

English

URL of your Intervention Website or App

e.g. a direct link to the mobile app on app in appstore (itunes, Google Play), or URL of the website. If the intervention is a DVD or hardware, you can also link to an Amazon page.

https://www.lezparlay.com

URL of an image/screenshot (optional)

Your answer

Your response is too large. Try shortening some answers.

Accessibility *

Can an enduser access the intervention presently?

- ○ access is free and open
- ◉ access only for special usergroups, not open
- ○ access is open to everyone, but requires payment/subscription/in-app purchases
- ○ app/intervention no longer accessible
- ○ Other:

Primary Medical Indication/Disease/Condition *

e.g. "Stress", "Diabetes", or define the target group in brackets after the condition, e.g. "Autism (Parents of children with)", "Alzheimers (Informal Caregivers of)"

Heavy Alcohol Use

Primary Outcomes measured in trial *

comma-separated list of primary outcomes reported in the trial

Alcohol Consumption, Negative Alcohol-Relate

Secondary/other outcomes

Are there any other outcomes the intervention is expected to affect?

Perceived peer drinking norms (mediator)

Your response is too large. Try shortening some answers.

Recommended "Dose" *

What do the instructions for users say on how often the app should be used?

- ○ Approximately Daily
- ○ Approximately Weekly
- ○ Approximately Monthly
- ○ Approximately Yearly
- ○ "as needed"
- ◉ Other: The app included 8 monthly rounds of a competition (in which an inte[illegible]

Approx. Percentage of Users (starters) still using the app as recommended after 3 months *

- ◉ unknown / not evaluated
- ○ 0-10%
- ○ 11-20%
- ○ 21-30%
- ○ 31-40%
- ○ 41-50%
- ○ 51-60%
- ○ 61-70%
- ○ 71%-80%
- ○ 81-90%
- ○ 91-100%
- ○ Other:

Your response is too large. Try shortening some answers.

Overall, was the app/intervention effective? *

- ○ yes: all primary outcomes were significantly better in intervention group vs control
- ○ partly: SOME primary outcomes were significantly better in intervention group vs control
- ○ no statistically significant difference between control and intervention
- ○ potentially harmful: control was significantly better than intervention in one or more outcomes
- ○ inconclusive: more research is needed
- ◉ Other: This is a protocol paper. Analysis is currently underway.

Article Preparation Status/Stage *

At which stage in your article preparation are you currently (at the time you fill in this form)

- ○ not submitted yet - in early draft status
- ○ not submitted yet - in late draft status, just before submission
- ○ submitted to a journal but not reviewed yet
- ◉ submitted to a journal and after receiving initial reviewer comments
- ○ submitted to a journal and accepted, but not published yet
- ○ published
- ○ Other:

Your response is too large. Try shortening some answers.

Journal *

If you already know where you will submit this paper (or if it is already submitted), please provide the journal name (if it is not JMIR, provide the journal name under "other")

- ○ not submitted yet / unclear where I will submit this
- ○ Journal of Medical Internet Research (JMIR)
- ○ JMIR mHealth and UHealth
- ○ JMIR Serious Games
- ○ JMIR Mental Health
- ○ JMIR Public Health
- ○ JMIR Formative Research
- ○ Other JMIR sister journal
- ◉ Other: JMIR Research Protocols

Is this a full powered effectiveness trial or a pilot/feasibility trial? *

- ○ Pilot/feasibility
- ◉ Fully powered

Manuscript tracking number *

If this is a JMIR submission, please provide the manuscript tracking number under "other" (The ms tracking number can be found in the submission acknowledgement email, or when you login as author in JMIR. If the paper is already published in JMIR, then the ms tracking number is the four-digit number at the end of the DOI, to be found at the bottom of each published article in JMIR)

- ○ no ms number (yet) / not (yet) submitted to / published in JMIR
- ◉ Other: JRP#24647

Your response is too large. Try shortening some answers.

TITLE AND ABSTRACT

1a) TITLE: Identification as a randomized trial in the title

1a) Does your paper address CONSORT item 1a? *

I.e does the title contain the phrase "Randomized Controlled Trial"? (if not, explain the reason under "other")

- ○ yes
- ◉ Other: The title contains the phrase "hybrid trial" because this is a type I effic

1a-i) Identify the mode of delivery in the title

Identify the mode of delivery. Preferably use "web-based" and/or "mobile" and/or "electronic game" in the title. Avoid ambiguous terms like "online", "virtual", "interactive". Use "Internet-based" only if Intervention includes non-web-based Internet components (e.g. email), use "computer-based" or "electronic" only if offline products are used. Use "virtual" only in the context of "virtual reality" (3-D worlds). Use "online" only in the context of "online support groups". Complement or substitute product names with broader terms for the class of products (such as "mobile" or "smart phone" instead of "iphone"), especially if the application runs on different platforms.

	1	2	3	4	5	
subitem not at all important	○	○	○	○	◉	essential

Clear selection

Does your paper address subitem 1a-i? *

Copy and paste relevant sections from manuscript title (include quotes in quotation marks "like this" to indicate direct quotes from your manuscript), or elaborate on this item by providing additional information not in the ms, or briefly explain why the item is not applicable/relevant for your study

"A Gamified, Social Media Inspired, Web-based Personalized Normative Feedback Alcohol Intervention for Lesbian, Bisexual, and Queer Identified Women: Protocol [illegible]

Your response is too large. Try shortening some answers.

1a-ii) Non-web-based components or important co-interventions in title

Mention non-web-based components or important co-interventions in title, if any (e.g., “with telephone support”).

	1	2	3	4	5	
subitem not at all important	◉	○	○	○	○	essential

Clear selection

Does your paper address subitem 1a-ii?

Copy and paste relevant sections from manuscript title (include quotes in quotation marks "like this" to indicate direct quotes from your manuscript), or elaborate on this item by providing additional information not in the ms, or briefly explain why the item is not applicable/relevant for your study

not applicable

1a-iii) Primary condition or target group in the title

Mention primary condition or target group in the title, if any (e.g., “for children with Type I Diabetes”) Example: A Web-based and Mobile Intervention with Telephone Support for Children with Type I Diabetes: Randomized Controlled Trial

	1	2	3	4	5	
subitem not at all important	○	○	○	○	◉	essential

Clear selection

Your response is too large. Try shortening some answers.

Does your paper address subitem 1a-iii? *

Copy and paste relevant sections from manuscript title (include quotes in quotation marks "like this" to indicate direct quotes from your manuscript), or elaborate on this item by providing additional information not in the ms, or briefly explain why the item is not applicable/relevant for your study

Yes. "A Gamified, Social Media Inspired, Web-based Personalized Normative Feedback Alcohol Intervention for Lesbian, Bisexual, and Queer Identified Women: Protocol for a Hybrid Trial"

1b) ABSTRACT: Structured summary of trial design, methods, results, and conclusions

NPT extension: Description of experimental treatment, comparator, care providers, centers, and blinding status.

1b-i) Key features/functionalities/components of the intervention and comparator in the METHODS section of the ABSTRACT

Mention key features/functionalities/components of the intervention and comparator in the abstract. If possible, also mention theories and principles used for designing the site. Keep in mind the needs of systematic reviewers and indexers by including important synonyms. (Note: Only report in the abstract what the main paper is reporting. If this information is missing from the main body of text, consider adding it)

	1	2	3	4	5	
subitem not at all important	○	○	○	○	◉	essential

Clear selection

Your response is too large. Try shortening some answers.

Does your paper address subitem 1b-i? *

Copy and paste relevant sections from the manuscript abstract (include quotes in quotation marks "like this" to indicate direct quotes from your manuscript), or elaborate on this item by providing additional information not in the ms, or briefly explain why the item is not applicable/relevant for your study

Your response is too large. Try shortening some answers.

Yes.
"BACKGROUND: Sexual minority women (SMW) are more likely to drink alcohol, engage in heavy drinking, and experience alcohol-related problems than are heterosexual women. Yet, to date, culturally tailored interventions for this population have been slow to emerge.

OBJECTIVE: This Type I Effectiveness/Implementation Trial examines the feasibility and efficacy of a gamified, culturally tailored, personalized normative feedback (PNF) alcohol intervention for SMW who psychologically identify as lesbian, bisexual, or queer (LBQ).

METHODS: The core components of a PNF intervention were delivered within LezParlay, a fun, social media inspired, digital competition designed to challenge negative stereotypes about lesbian, bisexual, and queer women and increase visibility. Following two rounds of play by a large cohort of SMW, a sub-sample of 500 drinkers already taking part in the competition were invited to participate in an evaluation study. Study participants were randomized to receive 1 of 3 unique sequences of PNF (i.e., Alcohol & Stigma-Coping, Alcohol & Control, or Control topics only) over 2 intervention rounds.

RESULTS: Analyses will evaluate whether PNF on alcohol use reduces participants' drinking and negative consequences 2 and 4 months post-intervention, examine whether providing PNF on stigma-coping behaviors in addition to alcohol use further reduces alcohol use and consequences beyond alcohol PNF alone, identify mediators and moderators of intervention efficacy, and examine broader LezParlay app engagement, acceptability, and perceived benefits.

CONCLUSION: This "incognito" intervention approach is uniquely oriented toward engaging and preventing alcohol-related risks among community populations of LBQ women who may view their heavy drinking as normative and not in need of change due to the visibility of alcohol use in sexual minority community spaces. Thus, the present intervention strategy diverges from, and is intended to compliment more intensive programs being developed to meet the needs of SMW already motivated to reduce their consumption."

Your response is too large. Try shortening some answers.

1b-ii) Level of human involvement in the METHODS section of the ABSTRACT

Clarify the level of human involvement in the abstract, e.g., use phrases like "fully automated" vs. "therapist/nurse/care provider/physician-assisted" (mention number and expertise of providers involved, if any). (Note: Only report in the abstract what the main paper is reporting. If this information is missing from the main body of text, consider adding it)

	1	2	3	4	5	
subitem not at all important	(●)	( )	( )	( )	( )	essential

Clear selection

Does your paper address subitem 1b-ii?

Copy and paste relevant sections from the manuscript abstract (include quotes in quotation marks "like this" to indicate direct quotes from your manuscript), or elaborate on this item by providing additional information not in the ms, or briefly explain why the item is not applicable/relevant for your study

N/A. This is a digital intervention with no researcher/clinician interaction or assistance

1b-iii) Open vs. closed, web-based (self-assessment) vs. face-to-face assessments in the METHODS section of the ABSTRACT

Mention how participants were recruited (online vs. offline), e.g., from an open access website or from a clinic or a closed online user group (closed usergroup trial), and clarify if this was a purely web-based trial, or there were face-to-face components (as part of the intervention or for assessment). Clearly say if outcomes were self-assessed through questionnaires (as common in web-based trials). Note: In traditional offline trials, an open trial (open-label trial) is a type of clinical trial in which both the researchers and participants know which treatment is being administered. To avoid confusion, use "blinded" or "unblinded" to indicated the level of blinding instead of "open", as "open" in web-based trials usually refers to "open access" (i.e. participants can self-enrol). (Note: Only report in the abstract what the main paper is reporting. If this information is missing from the main body of text, consider adding it)

	1	2	3	4	5	
subitem not at all important	(●)	( )	( )	( )	( )	essential

Clear selection

Your response is too large. Try shortening some answers.

Does your paper address subitem 1b-iii?

Copy and paste relevant sections from the manuscript abstract (include quotes in quotation marks "like this" to indicate direct quotes from your manuscript), or elaborate on this item by providing additional information not in the ms, or briefly explain why the item is not applicable/relevant for your study

Not applicable

1b-iv) RESULTS section in abstract must contain use data

Report number of participants enrolled/assessed in each group, the use/uptake of the intervention (e.g., attrition/adherence metrics, use over time, number of logins etc.), in addition to primary/secondary outcomes. (Note: Only report in the abstract what the main paper is reporting. If this information is missing from the main body of text, consider adding it)

	1	2	3	4	5	
subitem not at all important	◉	○	○	○	○	essential

Clear selection

Does your paper address subitem 1b-iv?

Copy and paste relevant sections from the manuscript abstract (include quotes in quotation marks "like this" to indicate direct quotes from your manuscript), or elaborate on this item by providing additional information not in the ms, or briefly explain why the item is not applicable/relevant for your study

This is a protocol paper. Analysis of data has not yet occurred.

Your response is too large. Try shortening some answers.

1b-v) CONCLUSIONS/DISCUSSION in abstract for negative trials

Conclusions/Discussions in abstract for negative trials: Discuss the primary outcome - if the trial is negative (primary outcome not changed), and the intervention was not used, discuss whether negative results are attributable to lack of uptake and discuss reasons. (Note: Only report in the abstract what the main paper is reporting. If this information is missing from the main body of text, consider adding it)

	1	2	3	4	5	
subitem not at all important	◉	○	○	○	○	essential

Clear selection

Does your paper address subitem 1b-v?

Copy and paste relevant sections from the manuscript abstract (include quotes in quotation marks "like this" to indicate direct quotes from your manuscript), or elaborate on this item by providing additional information not in the ms, or briefly explain why the item is not applicable/relevant for your study

This is a protocol paper. Analysis of data has not yet occurred.

INTRODUCTION

2a) In INTRODUCTION: Scientific background and explanation of rationale

2a-i) Problem and the type of system/solution

Describe the problem and the type of system/solution that is object of the study: intended as stand-alone intervention vs. incorporated in broader health care program? Intended for a particular patient population? Goals of the intervention, e.g., being more cost-effective to other interventions, replace or complement other solutions? (Note: Details about the intervention are provided in “Methods” under 5)

	1	2	3	4	5	
subitem not at all important	○	○	○	○	◉	essential

Your response is too large. Try shortening some answers.

Does your paper address subitem 2a-i? *

Copy and paste relevant sections from the manuscript (include quotes in quotation marks "like this" to indicate direct quotes from your manuscript), or elaborate on this item by providing additional information not in the ms, or briefly explain why the item is not applicable/relevant for your study

Yes.
"Why a Brief, Social Norms-Based Alcohol Intervention for SMW?
Although social norms are among the most predictive and commonly targeted antecedent to alcohol use in other heavy drinking populations [20–23] and a growing literature identifies sexual minority-specific peer substance use norms as appropriate targets for intervention and prevention efforts [14,15,24–27], the dominant perspective for understanding heavy drinking among SMW is not that of social norms, but rather that of sexual minority stress [28–30]. This model emphasizes the role of stigma in SMW's increased alcohol use, explaining that increased drinking and dependence among SMW may derive from separate and combined effects of distal stressors—including experiences of prejudice, rejection, harassment, discrimination, and violence originating from heterosexual society [28,30,31], as well as proximal stressors rooted within the individual including internalization of stigma, concealment, and inadequate or problematic coping. Growing support for the link between sexual minority stress exposure and coping-motivated drinking [32–38] have encouraged the development of intensive, sexual identity affirming programs that seek to reduce alcohol use and improve mental health by increasing individuals' understanding of stigma-related processes and bolstering their adaptive coping skills and resources [39,40]. Despite the promise of these interventions for SMW seeking treatment for their problematic drinking [41], these strategies are unlikely to engage the larger population of SMW who do not view their drinking as problematic or lack motivation to reduce their consumption. In contrast, social norms interventions have the potential to cost-effectively reach, and motivate reductions in drinking among SMW who do not yet view their drinking as excessive or a risk to their overall health. Further, much like the heavy drinking populations of college students [20,43], military personnel [21,22], and working adults [44] commonly targeted by PNF alcohol interventions, bisexual and lesbian-identified SMW have been found to overestimate descriptive peer drinking norms [13,14]. Over-time, these perceptions of norms have been found to relate to drinking behavior among lesbian and bisexual SMW in the standard reciprocal feed-forward fashion observed in other groups for whom PNF interventions have been effective [15]. Building on these findings, the primary aim of the present trial is to examine the extent to which PNF designed to correct sexual identity and age-specific [illegible]

Your response is too large. Try shortening some answers.

thereby reducing LBQ women's alcohol use and negative consequences. Recent research also suggests that in addition to standard frequency and quantity peer [illegible]

research also suggests that in addition to standard frequency and quantity peer drinking norms, this population also over-estimates peer norms specific to coping-motivated drinking following collectively experienced sexual minority stressors such as the Pulse Nightclub shooting [45] and 2016 U.S. Presidential election [46], with these misperceptions also contributing to their current and future drinking beyond the self-reported stress impacts of these events. As such, a secondary aim of this trial to is evaluate whether delivering PNF on stigma-coping behaviors in addition to alcohol use further reduces alcohol use and consequences beyond PNF on alcohol use alone."

2a-ii) Scientific background, rationale: What is known about the (type of) system

Scientific background, rationale: What is known about the (type of) system that is the object of the study (be sure to discuss the use of similar systems for other conditions/diagnoses, if appropiate), motivation for the study, i.e. what are the reasons for and what is the context for this specific study, from which stakeholder viewpoint is the study performed, potential impact of findings [2]. Briefly justify the choice of the comparator.

	1	2	3	4	5	
subitem not at all important	○	○	○	●	○	essential

Clear selection

Your response is too large. Try shortening some answers.

Does your paper address subitem 2a-ii? *

Copy and paste relevant sections from the manuscript (include quotes in quotation marks "like this" to indicate direct quotes from your manuscript), or elaborate on this item by providing additional information not in the ms, or briefly explain why the item is not applicable/relevant for your study

YES.

"Addressing PNF Intervention Limitations & Engaging a Hard to Reach Population

Extensive study of web-based PNF interventions among college students suggest that these interventions lead to reliable but relatively short-term and modest reductions in drinking [20]. Researchers have identified a number of issues that if remedied, could considerably increase the impact of this strategy. In particular, doubts about the credibility of actual drinking statistics presented [55,56], defensive reactions among heavy drinkers [57,58], general inattention to feedback [59,60], and low motivation among participants [61] have been proposed as barriers to greater public health impact. The real-world suitablity of this approach has also drawn criticism [62] as researchers have struggled to implement web-based PNF interventions as well as engage and retain heavy drinkers outside of study settings where participation is mandatory or participants are offerred compensation at the point of recruitment [61,63]. Beyond these issues, sexual minority communities also present their own unique challenges for intervention dissemination. Unlike universities and military bases where mandatory PNF interventions can easily target new cohorts of students and recruits, there is no single institution from which SMW can be as easily recruited. In contrast, SMW must hear about a community program and judge the program credible and worthwhile.

One tool commonly used to increase the credibility and appeal of health promotion programs for minority populations is cultural tailoring, which refers to the development of interventions, messages, and materials to conform with specific cultural characteristics of the target group [64]. Recommended cultural tailoring practices for SMW include the development of programs and materials that reflect the social identities, values, and lived experiences of LBQ women as well as involvement of LBQ community members and trusted community organizations in program promotion and delivery [12,65,66]. Following these recommendations and seeking to bolster intervention relevance, engagement, and motivation, PNF designed to correct drinking and stigma-coping norms were delivered within a larger digital competition called LezParlay. This competition was strategically crafted to reflect deep structure cultural themes including community [illegible] identity visibility [70–72], and enjoyment of intra-community competition and sport [illegible]

Your response is too large. Try shortening some answers.

[73–75]. Consistent with recommendations for surface-structure intervention tailoring [12,65,76], the LezParlay competition was also developed by an LBQ woman in the target age-range and jointly promoted online by five collaborating community organizations (i.e., HER Social App, Autostraddle, LezDoBrunch, the LA LGBT Center) trusted as sources of health and social information by LBQ women. In addition to cultural tailoring, LezParlay also draws upon on self-determination theory (SDT; [77,78]) and the nascent gamification literature [79–82] to leverage four evidence-based game mechanics (i.e., copresence, a system of points, user-generated content, chance-based uncertainty) to both remedy limitations associated with traditional PNF intervention formats and foster basic psychological needs for relatedness, competence, and autonomy in this population (See Multimedia Appendix for an overview of LezParlay game mechanics and supporting literature).

One round of LezParlay was played monthly over an 8 month period with a variable cash prize awarded monthly to the top scoring player exhibiting the greatest accuracy in their perceptions of LBQ peers. During the first 3 weeks of each round, players were invited to size-up fellow LBQ players by browsing their social-media like profiles, submit guesses about negative stereotype related behaviors and experiences of age and sexual identity matched LBQ peers' (e.g., What percent of [lesbian/bisexual/queer] players in their [20s/30s/40s/50s+] own a pair of Birkenstocks? How many days per week does the typical [lesbian/bisexual/queer] player in her [20s/30s/40s/50s+] drink?), select an amount of points to wager on these guesses being true of other age and sexual identity matched players, and earn points for reporting on their own corresponding behaviors and experiences. The last week of each month, all players received individualized detailed results (i.e., PNF) for a subset of the round's questions. Animated charts and text detailed the accuracy of the player's perceptions, how their behaviors and experiences compared to LBQ peers, summarized of the stereotypes challenged, and provided their perceptual-accuracy based rank and score. Importantly, all actual norms featured in detailed results were derived organically from players' round-specific reports of their behaviors and experiences. The Multimedia Appendix provides detailed descriptions of LezParlay round play and detailed results (i.e., PNF screens)."

2b) In INTRODUCTION: Specific objectives or hypotheses

Your response is too large. Try shortening some answers.

Does your paper address CONSORT subitem 2b? *

Copy and paste relevant sections from the manuscript (include quotes in quotation marks "like this" to indicate direct quotes from your manuscript), or elaborate on this item by providing additional information not in the ms, or briefly explain why the item is not applicable/relevant for your study

YES.
"The Current Study
This registered clinical trial seeks evaluate whether LezParlay delivered PNF on alcohol use corrects drinking norms and reduces alcohol consumption and negative consequences relative to PNF on control topics (AIM 1), examine whether providing PNF on coping behaviors in addition to alcohol use further reduces alcohol use and consequences beyond alcohol PNF alone (AIM 2), identify mediators (i.e., percieved norms) and moderators (i.e., interpersonal stigma exposure, baseline drinking, sexual identity, age, relationship status, race/ethnicity) of intervention efficacy (AIM 3), and examine broader LezParlay competition engagement, acceptability, and perceived benefits (AIM 4)."

METHODS

3a) Description of trial design (such as parallel, factorial) including allocation ratio

Your response is too large. Try shortening some answers.

Does your paper address CONSORT subitem 3a? *

Copy and paste relevant sections from the manuscript (include quotes in quotation marks "like this" to indicate direct quotes from your manuscript), or elaborate on this item by providing additional information not in the ms, or briefly explain why the item is not applicable/relevant for your study

Yes.
"Trial Design

Following recent recommendations for testing the real-world feasibility and impact of normative feedback interventions [62,83], LezParlay is examined through a Type I Hybrid-Effectiveness-Implementation Trial [84,85]. That is, in contrast to recruiting LBQ women into a transparent, incentivized, alcohol intervention study, LezParlay was advertised as it would be in the real-world— as a free, online competition designed to test LBQ stereotypes and increase visibility. Only after several rounds of play were a sub-sample of 500 drinkers already taking part in the competition invited to take part in an incentivized Evaluation Study. These players were covertly randomized to receive 1 of 3 unique sequences of feedback (i.e., Alcohol+Coping, Alcohol Only, or Control Only) over 2 consecutive rounds of play. Short-term reductions in norms and drinking were assessed 2 months later organically within the competition through a "Replay Bonus" which invited players to boost their scores by guessing, betting, and reporting on alcohol use and control topics a second time. Then, following the competition, 4 months post-intervention, Evaluation Study participants completed a feedback survey assessing competition acceptability, perceived benefits, and feature requests for the next version of the competition. At the end of this survey, participants reported their alcohol use a final time."

3b) Important changes to methods after trial commencement (such as eligibility criteria), with reasons

Your response is too large. Try shortening some answers.

Does your paper address CONSORT subitem 3b? *

Copy and paste relevant sections from the manuscript (include quotes in quotation marks "like this" to indicate direct quotes from your manuscript), or elaborate on this item by providing additional information not in the ms, or briefly explain why the item is not applicable/relevant for your study

No. This is a protocol paper submitted following data collection but prior to any analysis.

3b-i) Bug fixes, Downtimes, Content Changes

Bug fixes, Downtimes, Content Changes: ehealth systems are often dynamic systems. A description of changes to methods therefore also includes important changes made on the intervention or comparator during the trial (e.g., major bug fixes or changes in the functionality or content) (5-iii) and other “unexpected events” that may have influenced study design such as staff changes, system failures/downtimes, etc. [2].

	1	2	3	4	5	
subitem not at all important	(selected)	( )	( )	( )	( )	essential

Clear selection

Does your paper address subitem 3b-i?

Copy and paste relevant sections from the manuscript (include quotes in quotation marks "like this" to indicate direct quotes from your manuscript), or elaborate on this item by providing additional information not in the ms, or briefly explain why the item is not applicable/relevant for your study

No such changes or bugs occurred.

4a) Eligibility criteria for participants

Your response is too large. Try shortening some answers.

Does your paper address CONSORT subitem 4a? *

Copy and paste relevant sections from the manuscript (include quotes in quotation marks "like this" to indicate direct quotes from your manuscript), or elaborate on this item by providing additional information not in the ms, or briefly explain why the item is not applicable/relevant for your study

Yes.
"RCT Sub-Sample Recruitment. There was no upper limit on the number of SMW who could take part in LezParlay and new players were accepted on a rolling basis throughout the competition. We aimed to recruit a minimum of 1,200 LBQ women to sign-up during the first two monthly rounds to ensure that meaningful and stable sexual identity and age-group specific actual norms for drinking and coping behaviors could be delivered in intervention Rounds 3 and 4. From this larger pool of players, 500 drinkers were recruited to take part in a LezParlay Evaluation Study (RCT) during the third month of play. Acting as baseline (T1) for the RCT, Round 3 featured questions about alcohol use, stigma experiences, and a group of non-health related control questions submitted by players. Upon submitting answers to alcohol-related questions in Round 3, players were covertly screened for Evaluation Study eligibility based on their answers (i.e., number of drinking days per week and peak drinks on a single day during the past 2 months) as well as their geolocation and number of previous rounds played. Those who played at least 1 previous round, were located in the U.S., and reported drinking alcohol on 3 or more days per week OR having 3 or more drinks on their peak drinking occasion, were invited to take part in the Evaluation Study at the end of the round. Interested potential participants advanced to an informed consent screen which explained that the goal of the study was to evaluate the impact and format of detailed results received in LezParlay and gather player feedback to inform the next version of the competition. Information further detailed that participation in the Evaluation Study simply involved playing and viewing detailed results in subsequent rounds and completing a brief feedback survey at the end of the competition. Participants could earn up to $40 in e-gift cards of their choice for playing subsequent rounds and completing the feedback survey. Those who checked a box indicating that they understood what study participation entailed and desired to participate were welcomed into the study as LezParlay “official testers”."

Your response is too large. Try shortening some answers.

4a-i) Computer / Internet literacy

Computer / Internet literacy is often an implicit “de facto” eligibility criterion - this should be explicitly clarified.

	1	2	3	4	5	
subitem not at all important	(selected)	( )	( )	( )	( )	essential

Clear selection

Does your paper address subitem 4a-i?

Copy and paste relevant sections from the manuscript (include quotes in quotation marks "like this" to indicate direct quotes from your manuscript), or elaborate on this item by providing additional information not in the ms, or briefly explain why the item is not applicable/relevant for your study

Not applicable. The RCT sub-sample was recruited from a larger population already taking part in a broader web-based competition which they learned about through online advertisements. Thus, all RCT participants were internet literate and this did not have to be assessed.

4a-ii) Open vs. closed, web-based vs. face-to-face assessments:

Open vs. closed, web-based vs. face-to-face assessments: Mention how participants were recruited (online vs. offline), e.g., from an open access website or from a clinic, and clarify if this was a purely web-based trial, or there were face-to-face components (as part of the intervention or for assessment), i.e., to what degree got the study team to know the participant. In online-only trials, clarify if participants were quasi-anonymous and whether having multiple identities was possible or whether technical or logistical measures (e.g., cookies, email confirmation, phone calls) were used to detect/prevent these.

	1	2	3	4	5	
subitem not at all important	( )	(selected)	( )	( )	( )	essential

Clear selection

Your response is too large. Try shortening some answers.

Does your paper address subitem 4a-ii? *

Copy and paste relevant sections from the manuscript (include quotes in quotation marks "like this" to indicate direct quotes from your manuscript), or elaborate on this item by providing additional information not in the ms, or briefly explain why the item is not applicable/relevant for your study

"Competition Promotion

The competition was open to all LBQ women ages 21 years or older, regardless of birth sex. Players learned about LezParlay through 1 of 4 promotion strategies taking place over a 3-month period. First, prior to the launch of the first round, local SMW were invited to sign-up through flyers and promotional items distributed at LBQ community events in Los Angeles (i.e., a weekend brunch for queer women put organized by community group LezDoBrunch, a Queer Casino Night jointly put on by the Los Angeles LGBT Center and the Los Angeles Women's Network). Next, as the first round began, marketing campaigns on HER Social App, the leading dating/social app for LBQ women, invited users in their 3 largest markets (i.e., Los Angeles, New York City, and Chicago) to LezParlay via push notifications and in-app advertisements. An advertisement was also placed in the e-newsletter of Autostraddle, the leading independently owned news website for queer women. During the first 3 rounds of the competition, targeted campaigns on Facebook and Instagram also advertised LezParlay to LBQ women residing all over the United States. All recruitment materials linked to LezParlay's informational landing page [87] which presented an overview of the competition and provided a sign-up button that re-directed interested women to view and accept the Terms of Service and Privacy Policy (basic consent for competition participation) before creating an account. All recruitment materials, procedures, and intervention materials were approved by the Institutional Review Board at Loyola Marymount University (protocol # LMUIRB2018SU14).

Procedure

After consenting to take part in the competition, users were prompted to attach a valid mobile phone number to their account and could elect to login with a unique email address and password combination or use their existing Facebook credentials. Next, users created their LezParlay public profile which included a username of their choice and their sexual identity, age-group, relationship status, and pronouns. Users also had the option of uploading a profile photo or Bitmoji to represent them, entering a brief textual self-description, and connecting their Facebook, Twitter, and/or Instagram accounts so that other players could learn about them. Following account creation, players were directed to a home screen [illegible] buttons to play the current round, browse player profiles, submit and vote on [illegible]

Your response is too large. Try shortening some answers.

questions to be parlayed in future rounds, view round winners and leaderboards, edit public profile, and change account settings. The specifics of round play and the format of the detailed results (i.e., PNF) delivered at the end of each round are detailed in the Multimedia Appendix.

RCT Sub-Sample Recruitment. There was no upper limit on the number of SMW who could take part in LezParlay and new players were accepted on a rolling basis throughout the competition. We aimed to recruit a minimum of 1,200 LBQ women to sign-up during the first two monthly rounds to ensure that meaningful and stable sexual identity and age-group specific actual norms for drinking and coping behaviors could be delivered in intervention Rounds 3 and 4. From this larger pool of players, 500 drinkers were recruited to take part in a LezParlay Evaluation Study (RCT) during the third month of play. Acting as baseline (T1) for the RCT, Round 3 featured questions about alcohol use, stigma experiences, and a group of non-health related control questions submitted by players. Upon submitting answers to alcohol-related questions in Round 3, players were covertly screened for Evaluation Study eligibility based on their answers (i.e., number of drinking days per week and peak drinks on a single day during the past 2 months) as well as their geolocation and number of previous rounds played. Those who played at least 1 previous round, were located in the U.S., and reported drinking alcohol on 3 or more days per week OR having 3 or more drinks on their peak drinking occasion, were invited to take part in the Evaluation Study at the end of the round. Interested potential participants advanced to an informed consent screen which explained that the goal of the study was to evaluate the impact and format of detailed results received in LezParlay and gather player feedback to inform the next version of the competition. Information further detailed that participation in the Evaluation Study simply involved playing and viewing detailed results in subsequent rounds and completing a brief feedback survey at the end of the competition. Participants could earn up to $40 in e-gift cards of their choice for playing subsequent rounds and completing the feedback survey. Those who checked a box indicating that they understood what study participation entailed and desired to participate were welcomed into the study as LezParlay “official testers”."

Your response is too large. Try shortening some answers.

4a-iii) Information giving during recruitment

Information given during recruitment. Specify how participants were briefed for recruitment and in the informed consent procedures (e.g., publish the informed consent documentation as appendix, see also item X26), as this information may have an effect on user self-selection, user expectation and may also bias results.

	1	2	3	4	5	
subitem not at all important	(•)	( )	( )	( )	( )	essential

Clear selection

Does your paper address subitem 4a-iii?

Copy and paste relevant sections from the manuscript (include quotes in quotation marks "like this" to indicate direct quotes from your manuscript), or elaborate on this item by providing additional information not in the ms, or briefly explain why the item is not applicable/relevant for your study

Consent forms and the debriefing statement are publicly available on the clinical trial registration page: https://clinicaltrials.gov/ct2/show/NCT03884478

4b) Settings and locations where the data were collected

Does your paper address CONSORT subitem 4b? *

Copy and paste relevant sections from the manuscript (include quotes in quotation marks "like this" to indicate direct quotes from your manuscript), or elaborate on this item by providing additional information not in the ms, or briefly explain why the item is not applicable/relevant for your study

Yes. The method explains that all competition and RCT data were collected electronically by members of the research team located at Loyola Marymount University.

Your response is too large. Try shortening some answers.

4b-i) Report if outcomes were (self-)assessed through online questionnaires

Clearly report if outcomes were (self-)assessed through online questionnaires (as common in web-based trials) or otherwise.

	1	2	3	4	5	
subitem not at all important	○	○	○	○	◉	essential

Clear selection

Your response is too large. Try shortening some answers.

Does your paper address subitem 4b-i? *

Copy and paste relevant sections from the manuscript (include quotes in quotation marks "like this" to indicate direct quotes from your manuscript), or elaborate on this item by providing additional information not in the ms, or briefly explain why the item is not applicable/relevant for your study

Yes.
"RCT Measures

Demographic and Psychosocial Covariates. At sign-up, all players reported their sexual identity, relationship status, and age-group. Upon enrolling in the evaluation study participants also reported their race, ethnicity, and actual age in years. The feedback survey at the end of the competition prompted study participants to re-report their relationship status and sexual identity.

Perceived Alcohol-Related Norms and Behaviors. As described previously, perceived drinking norms and alcohol use behaviors were assessed organically in competition Rounds 3 (T1; baseline) and 7 (T2; 2-month follow-up) by items modeled after Baer's Quantity, Frequency, Max measure [88] in combination with additional norm and behavior items respectively examining negative alcohol-related consequences. These items were assessed a final time at the end of post-competition survey (T3; 4 month follow-up). Measures at each time-point referenced the previous 2-month period. As done in previous gamified PNF pilot studies with college students [43,89,90], composite measures of perceived alcohol-use norms and alcohol-use behavior at baseline and follow-up will be computed by z-scoring then averaging across respective sets of individual items at each time-point. In addition to these composites, 3 key outcomes of interest in alcohol intervention research are to be examined individually pre- and post- intervention: 1) estimated drinks per week over the previous 2 months (computed by multiplying reported number of drinking days per week and average number of drinks per occasion at each time-point), 2) peak drinks on one occasion over the previous 2 months, and, 3) number of negative alcohol-related consequences over the previous 2 months.

Interpersonal Stigma Exposure. Interpersonal stigma exposure was also assessed at baseline (Round 3) and follow-up (as a Replay Bonus topic in Round 7). Players guessed about the stigma experiences of other players and reported on their own stigma experiences over the previous 2 months. Stigma-related norms were not corrected in the competition and these perceptions were only assessed so that it made sense for players to report on their own recent interpersonal stigma exposure (a theorized moderator of conditional effects on drinking) at the same point in time that they were reporting on their alcohol use and negative [illegible]

Your response is too large. Try shortening some answers.

[illegible] stigma were assessed by their responses to 2 items (1) During the past 2 months, how many times have you been physically harmed due to your sexual identity? (2) [illegible]

how many times have you been physically harmed due to your sexual identity? (2) During the past 2 months, how many times have you been verbally harassed or threatened (online or in person) due to your sexual identity? Associations between pairs of stigma items at each time-point are expected and we anticipate combining responses to derive severe interpersonal stigma scores."

4b-ii) Report how institutional affiliations are displayed

Report how institutional affiliations are displayed to potential participants [on ehealth media], as affiliations with prestigious hospitals or universities may affect volunteer rates, use, and reactions with regards to an intervention.(Not a required item – describe only if this may bias results)

	1	2	3	4	5	
subitem not at all important	(●)	( )	( )	( )	( )	essential

Clear selection

Does your paper address subitem 4b-ii?

Copy and paste relevant sections from the manuscript (include quotes in quotation marks "like this" to indicate direct quotes from your manuscript), or elaborate on this item by providing additional information not in the ms, or briefly explain why the item is not applicable/relevant for your study

This was an incognito gamified intervention for a non-clinical/non-treatment seeking population. No university affiliation was presented at any point other than the very end of the RCT consent form where required.

5) The interventions for each group with sufficient details to allow replication, including how and when they were actually administered

Your response is too large. Try shortening some answers.

5-i) Mention names, credential, affiliations of the developers, sponsors, and owners

Mention names, credential, affiliations of the developers, sponsors, and owners [6] (if authors/evaluators are owners or developer of the software, this needs to be declared in a “Conflict of interest” section or mentioned elsewhere in the manuscript).

	1	2	3	4	5	
subitem not at all important	(●)	( )	( )	( )	( )	essential

Clear selection

Does your paper address subitem 5-i?

Copy and paste relevant sections from the manuscript (include quotes in quotation marks "like this" to indicate direct quotes from your manuscript), or elaborate on this item by providing additional information not in the ms, or briefly explain why the item is not applicable/relevant for your study

Yes.
"Funded by the National Institutes of Healths’ Exploratory/Developmental R21 grant mechanism, the goal of this initial trial is to evaluate the feasibility and efficacy associated with the LezParlay competition as “minimally viable product” taking the form of an extremely low-cost progressive web app designed and coded by the first author who is a member of the target population."

5-ii) Describe the history/development process

Describe the history/development process of the application and previous formative evaluations (e.g., focus groups, usability testing), as these will have an impact on adoption/use rates and help with interpreting results.

	1	2	3	4	5	
subitem not at all important	(●)	( )	( )	( )	( )	essential

Clear selection

Your response is too large. Try shortening some answers.

Does your paper address subitem 5-ii?

Copy and paste relevant sections from the manuscript (include quotes in quotation marks "like this" to indicate direct quotes from your manuscript), or elaborate on this item by providing additional information not in the ms, or briefly explain why the item is not applicable/relevant for your study

There was only ever a single version of the web app and it has not been previously evaluated in anyway.

5-iii) Revisions and updating

Revisions and updating. Clearly mention the date and/or version number of the application/intervention (and comparator, if applicable) evaluated, or describe whether the intervention underwent major changes during the evaluation process, or whether the development and/or content was “frozen” during the trial. Describe dynamic components such as news feeds or changing content which may have an impact on the replicability of the intervention (for unexpected events see item 3b).

	1	2	3	4	5	
subitem not at all important	(●)	( )	( )	( )	( )	essential

Clear selection

Your response is too large. Try shortening some answers.

Does your paper address subitem 5-iii?

Copy and paste relevant sections from the manuscript (include quotes in quotation marks "like this" to indicate direct quotes from your manuscript), or elaborate on this item by providing additional information not in the ms, or briefly explain why the item is not applicable/relevant for your study

Yes, there were 8 different rounds of questions and detailed results programmed in the app.

"One round of LezParlay was played monthly over an 8 month period with a variable cash prize awarded monthly to the top scoring player exhibiting the greatest accuracy in their perceptions of LBQ peers. During the first 3 weeks of each round, players were invited to size-up fellow LBQ players by browsing their social-media like profiles, submit guesses about negative stereotype related behaviors and experiences of age and sexual identity matched LBQ peers' (e.g., What percent of [lesbian/bisexual/queer] players in their [20s/30s/40s/50s+] own a pair of Birkenstocks? How many days per week does the typical [lesbian/bisexual/queer] player in her [20s/30s/40s/50s+] drink?), select an amount of points to wager on these guesses being true of other age and sexual identity matched players, and earn points for reporting on their own corresponding behaviors and experiences. The last week of each month, all players received individualized detailed results (i.e., PNF) for a subset of the round's questions. Animated charts and text detailed the accuracy of the player's perceptions, how their behaviors and experiences compared to LBQ peers, summarized of the stereotypes challenged, and provided their perceptual-accuracy based rank and score. Importantly, all actual norms featured in detailed results were derived organically from players' round-specific reports of their behaviors and experiences. The Multimedia Appendix provides detailed descriptions of LezParlay round play and detailed results (i.e., PNF screens)."

Your response is too large. Try shortening some answers.

5-iv) Quality assurance methods

Provide information on quality assurance methods to ensure accuracy and quality of information provided [1], if applicable.

	1	2	3	4	5	
subitem not at all important	(•)	( )	( )	( )	( )	essential

Clear selection

Does your paper address subitem 5-iv?

Copy and paste relevant sections from the manuscript (include quotes in quotation marks "like this" to indicate direct quotes from your manuscript), or elaborate on this item by providing additional information not in the ms, or briefly explain why the item is not applicable/relevant for your study

N/A

5-v) Ensure replicability by publishing the source code, and/or providing screenshots/screen-capture video, and/or providing flowcharts of the algorithms used

Ensure replicability by publishing the source code, and/or providing screenshots/screen-capture video, and/or providing flowcharts of the algorithms used. Replicability (i.e., other researchers should in principle be able to replicate the study) is a hallmark of scientific reporting.

	1	2	3	4	5	
subitem not at all important	(•)	( )	( )	( )	( )	essential

Clear selection

Your response is too large. Try shortening some answers.

Does your paper address subitem 5-v?

Copy and paste relevant sections from the manuscript (include quotes in quotation marks "like this" to indicate direct quotes from your manuscript), or elaborate on this item by providing additional information not in the ms, or briefly explain why the item is not applicable/relevant for your study

The multimedia appendix details functionality, provides screenshots of several app screens, and provides a web-based demo of the PNF intervention portion of the app.

5-vi) Digital preservation

Digital preservation: Provide the URL of the application, but as the intervention is likely to change or disappear over the course of the years; also make sure the intervention is archived (Internet Archive, webcitation.org, and/or publishing the source code or screenshots/videos alongside the article). As pages behind login screens cannot be archived, consider creating demo pages which are accessible without login.

	1	2	3	4	5	
subitem not at all important	○	○	○	○	◉	essential

Clear selection

Does your paper address subitem 5-vi?

Copy and paste relevant sections from the manuscript (include quotes in quotation marks "like this" to indicate direct quotes from your manuscript), or elaborate on this item by providing additional information not in the ms, or briefly explain why the item is not applicable/relevant for your study

app and demo URLs are cited as references.

Your response is too large. Try shortening some answers.

5-vii) Access

Access: Describe how participants accessed the application, in what setting/context, if they had to pay (or were paid) or not, whether they had to be a member of specific group. If known, describe how participants obtained "access to the platform and Internet" [1]. To ensure access for editors/reviewers/readers, consider to provide a "backdoor" login account or demo mode for reviewers/readers to explore the application (also important for archiving purposes, see vi).

	1	2	3	4	5	
subitem not at all important	(●)	( )	( )	( )	( )	essential

Clear selection

Does your paper address subitem 5-vii? *

Copy and paste relevant sections from the manuscript (include quotes in quotation marks "like this" to indicate direct quotes from your manuscript), or elaborate on this item by providing additional information not in the ms, or briefly explain why the item is not applicable/relevant for your study

This method and procedure clearly detail this. The app still exists and reviewers and readers may create accounts in order to check it out. However, the larger competition within which intervention content was embedded is over.

5-viii) Mode of delivery, features/functionalities/components of the intervention and comparator, and the theoretical framework

Describe mode of delivery, features/functionalities/components of the intervention and comparator, and the theoretical framework [6] used to design them (instructional strategy [1], behaviour change techniques, persuasive features, etc., see e.g., [7, 8] for terminology). This includes an in-depth description of the content (including where it is coming from and who developed it) [1]," whether [and how] it is tailored to individual circumstances and allows users to track their progress and receive feedback" [6]. This also includes a description of communication delivery channels and – if computer-mediated communication is a component – whether communication was synchronous or asynchronous [6]. It also includes information on presentation strategies [1], including page design principles, average amount of text on pages, presence of hyperlinks to other resources, etc. [1].

	1	2	3	4	5	
subitem not at all important	( )	( )	( )	( )	( )	essential

Your response is too large. Try shortening some answers.

Does your paper address subitem 5-viii? *

Copy and paste relevant sections from the manuscript (include quotes in quotation marks "like this" to indicate direct quotes from your manuscript), or elaborate on this item by providing additional information not in the ms, or briefly explain why the item is not applicable/relevant for your study

and procedure. app specific features of this is described in the multimedia appendix

5-ix) Describe use parameters

Describe use parameters (e.g., intended “doses” and optimal timing for use). Clarify what instructions or recommendations were given to the user, e.g., regarding timing, frequency, heaviness of use, if any, or was the intervention used ad libitum.

	1	2	3	4	5	
subitem not at all important	◉	○	○	○	○	essential

Clear selection

Does your paper address subitem 5-ix?

Copy and paste relevant sections from the manuscript (include quotes in quotation marks "like this" to indicate direct quotes from your manuscript), or elaborate on this item by providing additional information not in the ms, or briefly explain why the item is not applicable/relevant for your study

Not applicable. Rounds of the competition were played monthly and feedback on intervention/non-intervention topics were delivered by text message at the end of each month.

Your response is too large. Try shortening some answers.

5-x) Clarify the level of human involvement

Clarify the level of human involvement (care providers or health professionals, also technical assistance) in the e-intervention or as co-intervention (detail number and expertise of professionals involved, if any, as well as “type of assistance offered, the timing and frequency of the support, how it is initiated, and the medium by which the assistance is delivered”. It may be necessary to distinguish between the level of human involvement required for the trial, and the level of human involvement required for a routine application outside of a RCT setting (discuss under item 21 – generalizability).

	1	2	3	4	5	
subitem not at all important	◉	○	○	○	○	essential

Clear selection

Does your paper address subitem 5-x?

Copy and paste relevant sections from the manuscript (include quotes in quotation marks "like this" to indicate direct quotes from your manuscript), or elaborate on this item by providing additional information not in the ms, or briefly explain why the item is not applicable/relevant for your study

There was no such human involvement in this trial.

5-xi) Report any prompts/reminders used

Report any prompts/reminders used: Clarify if there were prompts (letters, emails, phone calls, SMS) to use the application, what triggered them, frequency etc. It may be necessary to distinguish between the level of prompts/reminders required for the trial, and the level of prompts/reminders for a routine application outside of a RCT setting (discuss under item 21 – generalizability).

	1	2	3	4	5	
subitem not at all important	○	○	○	○	◉	essential

Clear selection

Your response is too large. Try shortening some answers.

Does your paper address subitem 5-xi? *

Copy and paste relevant sections from the manuscript (include quotes in quotation marks "like this" to indicate direct quotes from your manuscript), or elaborate on this item by providing additional information not in the ms, or briefly explain why the item is not applicable/relevant for your study

Players taking part in the larger competition not taking part in the RCT and participants taking part in the RCT in all study conditions received the same automated SMS and email messages at the end of each month. These messages provided each user a personalized URL at which they could view detailed results and were uniform at the end of each round as follows:
"Hey [username], your detailed results from LezParlay Round [X1] are now ready to view! Check them out at [URL]. Round [X2] is also now open!

5-xii) Describe any co-interventions (incl. training/support)

Describe any co-interventions (incl. training/support): Clearly state any interventions that are provided in addition to the targeted eHealth intervention, as ehealth intervention may not be designed as stand-alone intervention. This includes training sessions and support [1]. It may be necessary to distinguish between the level of training required for the trial, and the level of training for a routine application outside of a RCT setting (discuss under item 21 – generalizability.

	1	2	3	4	5	
subitem not at all important	◉	○	○	○	○	essential

Clear selection

Does your paper address subitem 5-xii? *

Copy and paste relevant sections from the manuscript (include quotes in quotation marks "like this" to indicate direct quotes from your manuscript), or elaborate on this item by providing additional information not in the ms, or briefly explain why the item is not applicable/relevant for your study

Not applicable. No co-interventions.

Your response is too large. Try shortening some answers.

6a) Completely defined pre-specified primary and secondary outcome measures, including how and when they were assessed

Your response is too large. Try shortening some answers.

Does your paper address CONSORT subitem 6a? *

Copy and paste relevant sections from the manuscript (include quotes in quotation marks "like this" to indicate direct quotes from your manuscript), or elaborate on this item by providing additional information not in the ms, or briefly explain why the item is not applicable/relevant for your study

Yes.
"RCT Sub-Sample Recruitment. There was no upper limit on the number of SMW who could take part in LezParlay and new players were accepted on a rolling basis throughout the competition. We aimed to recruit a minimum of 1,200 LBQ women to sign-up during the first two monthly rounds to ensure that meaningful and stable sexual identity and age-group specific actual norms for drinking and coping behaviors could be delivered in intervention Rounds 3 and 4. From this larger pool of players, 500 drinkers were recruited to take part in a LezParlay Evaluation Study (RCT) during the third month of play. Acting as baseline (T1) for the RCT, Round 3 featured questions about alcohol use, stigma experiences, and a group of non-health related control questions submitted by players. Upon submitting answers to alcohol-related questions in Round 3, players were covertly screened for Evaluation Study eligibility based on their answers (i.e., number of drinking days per week and peak drinks on a single day during the past 2 months) as well as their geolocation and number of previous rounds played. Those who played at least 1 previous round, were located in the U.S., and reported drinking alcohol on 3 or more days per week OR having 3 or more drinks on their peak drinking occasion, were invited to take part in the Evaluation Study at the end of the round. Interested potential participants advanced to an informed consent screen which explained that the goal of the study was to evaluate the impact and format of detailed results received in LezParlay and gather player feedback to inform the next version of the competition. Information further detailed that participation in the Evaluation Study simply involved playing and viewing detailed results in subsequent rounds and completing a brief feedback survey at the end of the competition. Participants could earn up to $40 in e-gift cards of their choice for playing subsequent rounds and completing the feedback survey. Those who checked a box indicating that they understood what study participation entailed and desired to participate were welcomed into the study as LezParlay “official testers”.

RCT Design, Randomization, & Debriefing. The web app’s Qualtrics integration with ensured that Qualtrics Research Suite’s automated randomizer, commonly used in RCTs evaluating psychosocial interventions [40,41,55], could be used to randomize evaluation study participants to a PNF condition at the point of study enrollment in Round 3. Randomization determined the sequence of topics on which [illegible] Alcohol+Coping, Alcohol+Control, or Control Only. Members of the research team were blinded to participant condition assignment, and study participants were not

Your response is too large. Try shortening some answers.

were blinded to participant condition assignment, and study participants were not aware that any sort of randomization was taking place. Rather, when detailed results were sent at the end of each round, players were prompted to choose between doors in order to determine the 1-2 round topics on which they would view detailed results (See Figure 2 in the Multimedia Appendix). Although results topics were truly determined via chance in most rounds of the competition, the doors of Evaluation Study participants were "fixed" to open to their randomly assigned feedback topics regardless of the door they selected in Rounds 3 and 4. Upon completing the feedback survey at the end of the competition, participants were debriefed regarding the study's research questions and the fixed sequences of health or control feedback they were randomized to receive in Rounds 3 and 4 of the competition.

Intervention Rounds. All players taking part in the 3rd round of LezParlay estimated the drinking behaviors of the typical, same-sexual identity player in their age group during the previous 2 months, reporting on their perceptions of this typical player's (1) maximum number of drinks consumed on a single occasion, (2) average number of drinks consumed per occasion, and (3) average number of drinking days per week [83]. Players also estimated the number of negative-alcohol related consequences experienced over the previous 2 months by the typical player in their sexual identity and age-group from a list of 8 negative consequences (i.e., had a hangover or illness, got in a physical or verbal fight, had problems with significant other, missed a social engagement or event, had problems with friends or family, performed poorly at work or school, had an unwanted or regrettable sexual experience). Then, players answered parallel items assessing their own drinking and consequences over the corresponding 2-month period.

Players taking part in Round 4 of the competition were prompted to think about how other players deal with stress and sexual minority stigma and asked to estimate the percent of time (i.e., 0-100%) the typical player in their sexual identity and age-group tried to feel better during the past month by: (1) drinking alcohol; (2) taking a drug; (3) meditating, using relaxation techniques, or exercising; and, (4) talking to a close other or mental health professional. Then, players were prompted to think about how they, themselves, deal with stress and stigma and respond to parallel items. The actual norms variably delivered to evaluation study participants in PNF at the end of Rounds 3 and 4 were derived by computing the actual average response among all players submitting responses in each sexual identity and age-group.

Participants randomized to receive PNF on control topics received detailed results for non-health related topics in Round 3 (e.g., household repair ability, frequency of [illegible]

Your response is too large. Try shortening some answers.

Multimedia Appendix includes a link to view example detailed results for 1 control

topic and 1 treatment topic delivered in intervention rounds.

RCT Measures

Demographic and Psychosocial Covariates. At sign-up, all players reported their sexual identity, relationship status, and age-group. Upon enrolling in the evaluation study participants also reported their race, ethnicity, and actual age in years. The feedback survey at the end of the competition prompted study participants to re-report their relationship status and sexual identity.

Perceived Alcohol-Related Norms and Behaviors. As described previously, perceived drinking norms and alcohol use behaviors were assessed organically in competition Rounds 3 (T1; baseline) and 7 (T2; 2-month follow-up) by items modeled after Baer's Quantity, Frequency, Max measure [88] in combination with additional norm and behavior items respectively examining negative alcohol-related consequences. These items were assessed a final time at the end of post-competition survey (T3; 4 month follow-up). Measures at each time-point referenced the previous 2-month period. As done in previous gamified PNF pilot studies with college students [43,89,90], composite measures of perceived alcohol-use norms and alcohol-use behavior at baseline and follow-up will be computed by z-scoring then averaging across respective sets of individual items at each time-point. In addition to these composites, 3 key outcomes of interest in alcohol intervention research are to be examined individually pre- and post- intervention: 1) estimated drinks per week over the previous 2 months (computed by multiplying reported number of drinking days per week and average number of drinks per occasion at each time-point), 2) peak drinks on one occasion over the previous 2 months, and, 3) number of negative alcohol-related consequences over the previous 2 months.

Interpersonal Stigma Exposure. Interpersonal stigma exposure was also assessed at baseline (Round 3) and follow-up (as a Replay Bonus topic in Round 7). Players guessed about the stigma experiences of other players and reported on their own stigma experiences over the previous 2 months. Stigma-related norms were not corrected in the competition and these perceptions were only assessed so that it made sense for players to report on their own recent interpersonal stigma exposure (a theorized moderator of conditional effects on drinking) at the same point in time that they were reporting on their alcohol use and negative consequences in the game. Players' recent exposure to severe interpersonal stigma were assessed by their responses to 2 items (1) During the past 2 months, how many times have you been physically harmed due to your sexual identity? (2) During the past 2 months, how many times have you been verbally harassed or threatened (online or in person) due to your sexual identity? Associations between

Your response is too large. Try shortening some answers.

Feasibility Measures

Reach and Engagement. Data from Google Analytics and the application's backend will allow us to examine the total number of players who signed up to take part in the LezParlay competition in the absence of traditional study participation incentives, identify the promotional channels that brought them to the app, and detail players' demographic characteristics, states of residence, average number of logins, and number of rounds completed.

Acceptability. Feedback surveys prompted study participants to rate numerous aspects of the competition (the stereotype challenge concept, topics and questions, detailed results, leaderboards, the ability to browse player profiles, the ability to submit questions, the ability to bet points on the accuracy of guesses, text message and email communications from LezParlay) on Likert type scales ranging from did not like at all (0) to liked very much (5).

Perceived Benefits. A single Yes/No item asked participants whether they felt that participating in the LezParlay competition was psychologically beneficial. Those selecting 'yes' in response were asked to enter text describing perceived benefits.

Improvements & Requested Features. A final free response item asked participants to share recommendations they had for improving the competition and describe features they would like to see in the next version."

6a-i) Online questionnaires: describe if they were validated for online use and apply CHERRIES items to describe how the questionnaires were designed/deployed

If outcomes were obtained through online questionnaires, describe if they were validated for online use and apply CHERRIES items to describe how the questionnaires were designed/deployed [9].

	1	2	3	4	5	
subitem not at all important	(●)	○	○	○	○	essential

Clear selection

Your response is too large. Try shortening some answers.

Does your paper address subitem 6a-i?

Copy and paste relevant sections from manuscript text

RCT outcome measures (alcohol use, negative consequences) were modeled after previously published measures commonly assessed in online surveys. Additional feedback survey items (acceptability of competition aspects, open ended questions assessing perceived benefits and feature requests) were specific to the present intervention experience and were not used in any previous research.

"Perceived Alcohol-Related Norms and Behaviors. As described previously, perceived drinking norms and alcohol use behaviors were assessed organically in competition Rounds 3 (T1; baseline) and 7 (T2; 2-month follow-up) by items modeled after Baer's Quantity, Frequency, Max measure [88] in combination with additional norm and behavior items respectively examining negative alcohol-related consequences. These items were assessed a final time at the end of post-competition survey (T3; 4 month follow-up). Measures at each time-point referenced the previous 2-month period. As done in previous gamified PNF pilot studies with college students [43,89,90], composite measures of perceived alcohol-use norms and alcohol-use behavior at baseline and follow-up will be computed by z-scoring then averaging across respective sets of individual items at each time-point. In addition to these composites, 3 key outcomes of interest in alcohol intervention research are to be examined individually pre- and post- intervention: 1) estimated drinks per week over the previous 2 months (computed by multiplying reported number of drinking days per week and average number of drinks per occasion at each time-point), 2) peak drinks on one occasion over the previous 2 months, and, 3) number of negative alcohol-related consequences over the previous 2 months."

Your response is too large. Try shortening some answers.

6a-ii) Describe whether and how "use" (including intensity of use/dosage) was defined/measured/monitored

Describe whether and how "use" (including intensity of use/dosage) was defined/measured/monitored (logins, logfile analysis, etc.). Use/adoption metrics are important process outcomes that should be reported in any ehealth trial.

	1	2	3	4	5	
subitem not at all important	○	○	○	○	◉	essential

Clear selection

Does your paper address subitem 6a-ii?

Copy and paste relevant sections from manuscript text

Yes.
"Reach and Engagement. Data from Google Analytics and the application's backend will allow us to examine the total number of players who signed up to take part in the LezParlay competition in the absence of traditional study participation incentives, identify the promotional channels that brought them to the app, and detail players' demographic characteristics, states of residence, average number of logins, and number of rounds completed."

6a-iii) Describe whether, how, and when qualitative feedback from participants was obtained

Describe whether, how, and when qualitative feedback from participants was obtained (e.g., through emails, feedback forms, interviews, focus groups).

	1	2	3	4	5	
subitem not at all important	○	○	○	○	○	essential

Your response is too large. Try shortening some answers.

Does your paper address subitem 6a-iii?

Copy and paste relevant sections from manuscript text

Yes.
"

6b) Any changes to trial outcomes after the trial commenced, with reasons

Does your paper address CONSORT subitem 6b? *

Copy and paste relevant sections from the manuscript (include quotes in quotation marks "like this" to indicate direct quotes from your manuscript), or elaborate on this item by providing additional information not in the ms, or briefly explain why the item is not applicable/relevant for your study

"...following the competition, 4 months post-intervention, Evaluation Study participants completed a feedback survey assessing competition acceptability, percieved benefits, and feature requests for the next version of the competition. At the end of this survey, participants reported their alcohol use a final time."

7a) How sample size was determined

NPT: When applicable, details of whether and how the clustering by care provides or centers was addressed

7a-i) Describe whether and how expected attrition was taken into account when calculating the sample size

Describe whether and how expected attrition was taken into account when calculating the sample size.

	1	2	3	4	5	
subitem not at all important	○	○	○	○	◉	essential

Clear selection

Your response is too large. Try shortening some answers.

Does your paper address subitem 7a-i?

Copy and paste relevant sections from manuscript title (include quotes in quotation marks "like this" to indicate direct quotes from your manuscript), or elaborate on this item by providing additional information not in the ms, or briefly explain why the item is not applicable/relevant for your study

Yes.
"Power Analysis. Informed by previous research examining the effects of web-based alcohol PNF on changes in normative perceptions and drinking in other populations (d = .22; [20,99]), the comparably larger effect size revealed in a similar gamified PNF intervention for college students (d = .46; [89]), power analyses using the standard .80 power of detecting a significant effect, p < .05, and an effect size of d = .30 indicate a sample size of 375 (125 participants in each condition) to be sufficient to detect small-to-medium effects using repeated measures multi-level models (i.e. 2-levels, 3 arms, randomization at the individual level) as well as tests of mediation and moderation. Thus, our sample size of 500 will allow us to detect modest effects with even 30% attrition."

7b) When applicable, explanation of any interim analyses and stopping guidelines

Does your paper address CONSORT subitem 7b? *

Copy and paste relevant sections from the manuscript (include quotes in quotation marks "like this" to indicate direct quotes from your manuscript), or elaborate on this item by providing additional information not in the ms, or briefly explain why the item is not applicable/relevant for your study

N/A

8a) Method used to generate the random allocation sequence

NPT: When applicable, how care providers were allocated to each trial group

Your response is too large. Try shortening some answers.

Does your paper address CONSORT subitem 8a? *

Copy and paste relevant sections from the manuscript (include quotes in quotation marks "like this" to indicate direct quotes from your manuscript), or elaborate on this item by providing additional information not in the ms, or briefly explain why the item is not applicable/relevant for your study

There were no care providers involved. The intervention was automated through web app.

8b) Type of randomisation; details of any restriction (such as blocking and block size)

Does your paper address CONSORT subitem 8b? *

Copy and paste relevant sections from the manuscript (include quotes in quotation marks "like this" to indicate direct quotes from your manuscript), or elaborate on this item by providing additional information not in the ms, or briefly explain why the item is not applicable/relevant for your study

Simple randomization occurred via Qualtrics Research Suite's automated randomizer (web app contained a Qualtrics plugin).

9) Mechanism used to implement the random allocation sequence (such as sequentially numbered containers), describing any steps taken to conceal the sequence until interventions were assigned

Does your paper address CONSORT subitem 9? *

Copy and paste relevant sections from the manuscript (include quotes in quotation marks "like this" to indicate direct quotes from your manuscript), or elaborate on this item by providing additional information not in the ms, or briefly explain why the item is not applicable/relevant for your study

N/A. See previous response.

Your response is too large. Try shortening some answers.

10) Who generated the random allocation sequence, who enrolled participants, and who assigned participants to interventions

Your response is too large. Try shortening some answers.

Does your paper address CONSORT subitem 10? *

Copy and paste relevant sections from the manuscript (include quotes in quotation marks "like this" to indicate direct quotes from your manuscript), or elaborate on this item by providing additional information not in the ms, or briefly explain why the item is not applicable/relevant for your study

The web app did everything. All of this was automated.
"RCT Sub-Sample Recruitment. There was no upper limit on the number of SMW who could take part in LezParlay and new players were accepted on a rolling basis throughout the competition. We aimed to recruit a minimum of 1,200 LBQ women to sign-up during the first two monthly rounds to ensure that meaningful and stable sexual identity and age-group specific actual norms for drinking and coping behaviors could be delivered in intervention Rounds 3 and 4. From this larger pool of players, 500 drinkers were recruited to take part in a LezParlay Evaluation Study (RCT) during the third month of play. Acting as baseline (T1) for the RCT, Round 3 featured questions about alcohol use, stigma experiences, and a group of non-health related control questions submitted by players. Upon submitting answers to alcohol-related questions in Round 3, players were covertly screened for Evaluation Study eligibility based on their answers (i.e., number of drinking days per week and peak drinks on a single day during the past 2 months) as well as their geolocation and number of previous rounds played. Those who played at least 1 previous round, were located in the U.S., and reported drinking alcohol on 3 or more days per week OR having 3 or more drinks on their peak drinking occasion, were invited to take part in the Evaluation Study at the end of the round. Interested potential participants advanced to an informed consent screen which explained that the goal of the study was to evaluate the impact and format of detailed results received in LezParlay and gather player feedback to inform the next version of the competition. Information further detailed that participation in the Evaluation Study simply involved playing and viewing detailed results in subsequent rounds and completing a brief feedback survey at the end of the competition. Participants could earn up to $40 in e-gift cards of their choice for playing subsequent rounds and completing the feedback survey. Those who checked a box indicating that they understood what study participation entailed and desired to participate were welcomed into the study as LezParlay “official testers”.

RCT Design, Randomization, & Debriefing. The web app’s Qualtrics integration with ensured that Qualtrics Research Suite’s automated randomizer, commonly used in RCTs evaluating psychosocial interventions [40,41,55], could be used to randomize evaluation study participants to a PNF condition at the point of study enrollment in Round 3. Randomization determined the sequence of topics on which [illegible]

Your response is too large. Try shortening some answers.

[illegible] Alcohol+Coping, Alcohol+Control, or Control Only. Members of the research team were blinded to participant condition assignment, and study participants were not [illegible]

were blinded to participant condition assignment, and study participants were not aware that any sort of randomization was taking place. Rather, when detailed results were sent at the end of each round, players were prompted to choose between doors in order to determine the 1-2 round topics on which they would view detailed results (See Figure 2 in the Multimedia Appendix). Although results topics were truly determined via chance in most rounds of the competition, the doors of Evaluation Study participants were “fixed” to open to their randomly assigned feedback topics regardless of the door they selected in Rounds 3 and 4. Upon completing the feedback survey at the end of the competition, participants were debriefed regarding the study’s research questions and the fixed sequences of health or control feedback they were randomized to receive in Rounds 3 and 4 of the competition."

11a) If done, who was blinded after assignment to interventions (for example, participants, care providers, those assessing outcomes) and how

NPT: Whether or not administering co-interventions were blinded to group assignment

11a-i) Specify who was blinded, and who wasn’t

Specify who was blinded, and who wasn’t. Usually, in web-based trials it is not possible to blind the participants [1, 3] (this should be clearly acknowledged), but it may be possible to blind outcome assessors, those doing data analysis or those administering co-interventions (if any).

	1	2	3	4	5	
subitem not at all important	○	○	○	○	◉	essential

Clear selection

Your response is too large. Try shortening some answers.

Does your paper address subitem 11a-i? *

Copy and paste relevant sections from the manuscript (include quotes in quotation marks "like this" to indicate direct quotes from your manuscript), or elaborate on this item by providing additional information not in the ms, or briefly explain why the item is not applicable/relevant for your study

Both participants and members of the research team were blinded to study condition during the 8 month period that spanned the competition. Note that this was an incognito intervention embedded in a broader activity which contained several chance game mechanics. Thus, participants were not aware that any sort of randomization was taking place because the detailed results (PNF treatment) received across intervention rounds and non intervention rounds appeared to be based on these chance elements. Following all data collection, participant study condition assignment was unblinded for data analysis.

11a-ii) Discuss e.g., whether participants knew which intervention was the “intervention of interest” and which one was the “comparator”

Informed consent procedures (4a-ii) can create biases and certain expectations - discuss e.g., whether participants knew which intervention was the “intervention of interest” and which one was the “comparator”.

	1	2	3	4	5	
subitem not at all important	(●)	( )	( )	( )	( )	essential

Clear selection

Does your paper address subitem 11a-ii?

Copy and paste relevant sections from the manuscript (include quotes in quotation marks "like this" to indicate direct quotes from your manuscript), or elaborate on this item by providing additional information not in the ms, or briefly explain why the item is not applicable/relevant for your study

They did not know. See previous response.

Your response is too large. Try shortening some answers.

11b) If relevant, description of the similarity of interventions

(this item is usually not relevant for ehealth trials as it refers to similarity of a placebo or sham intervention to a active medication/intervention)

Your response is too large. Try shortening some answers.

Does your paper address CONSORT subitem 11b? *

Copy and paste relevant sections from the manuscript (include quotes in quotation marks "like this" to indicate direct quotes from your manuscript), or elaborate on this item by providing additional information not in the ms, or briefly explain why the item is not applicable/relevant for your study

Yes.
"Intervention Rounds. All players taking part in the 3rd round of LezParlay estimated the drinking behaviors of the typical, same-sexual identity player in their age group during the previous 2 months, reporting on their perceptions of this typical player's (1) maximum number of drinks consumed on a single occasion, (2) average number of drinks consumed per occasion, and (3) average number of drinking days per week [83]. Players also estimated the number of negative-alcohol related consequences experienced over the previous 2 months by the typical player in their sexual identity and age-group from a list of 8 negative consequences (i.e., had a hangover or illness, got in a physical or verbal fight, had problems with significant other, missed a social engagement or event, had problems with friends or family, performed poorly at work or school, had an unwanted or regrettable sexual experience). Then, players answered parallel items assessing their own drinking and consequences over the corresponding 2-month period.
Players taking part in Round 4 of the competition were prompted to think about how other players deal with stress and sexual minority stigma and asked to estimate the percent of time (i.e., 0-100%) the typical player in their sexual identity and age-group tried to feel better during the past month by: (1) drinking alcohol; (2) taking a drug; (3) meditating, using relaxation techniques, or exercising; and, (4) talking to a close other or mental health professional. Then, players were prompted to think about how they, themselves, deal with stress and stigma and respond to parallel items. The actual norms variably delivered to evaluation study participants in PNF at the end of Rounds 3 and 4 were derived by computing the actual average response among all players submitting responses in each sexual identity and age-group.
Participants randomized to receive PNF on control topics received detailed results for non-health related topics in Round 3 (e.g., household repair ability, frequency of home improvement store visits, tool box ownership, etc.) and Round 4 (e.g., time in between relationships, texting exes, partners being confused for sisters, etc.). The Multimedia Appendix includes a link to view example detailed results for 1 control topic and 1 treatment topic delivered in intervention rounds."

Your response is too large. Try shortening some answers.

12a) Statistical methods used to compare groups for primary and secondary outcomes

NPT: When applicable, details of whether and how the clustering by care providers or centers was addressed

Your response is too large. Try shortening some answers.

Does your paper address CONSORT subitem 12a? *

Copy and paste relevant sections from the manuscript (include quotes in quotation marks "like this" to indicate direct quotes from your manuscript), or elaborate on this item by providing additional information not in the ms, or briefly explain why the item is not applicable/relevant for your study

Yes.

"Data Analytic Plan for Evaluating Intervention Efficacy

An intent-to-treat approach will be used to examine LezParlay treatment effects at 2 and 4 months post-intervention on four outcomes: composite alcohol use, estimated number of drinks per week, peak number of drinks on one occasion, and number of negative alcohol-related consequences. Preliminary analyses will examine potential biases related to attrition and missing data [91,92], inspect outcome distributions, and evaluate potential baseline differences between conditions. As the latter 3 outcomes are count variables (i.e., estimated drinks per week, peak drinks, negative consequences) they are likely to be substantially skewed and best approximated by either Poission or Negative binomial distributions.

Main Effects. Two and four months following delivery of treatment PNF, participants in both conditions receiving treatment PNF on alcohol use (i.e., alcohol+coping, alcohol only) are expected to report reduced drinks per week, peak drinks, and negative consequences relative to those in the control PNF condition. Further, participants in the alcohol+coping condition are expected to exhibit larger reductions in their alcohol use and negative consequences at post-intervention follow-ups than participants in the alcohol only PNF condition. Multi-level models (MLMs [93,94]) with full maximum likelihood specification will be used to test these predictions. Time will be specified as a Level 1 varying predictor nested within individuals (Level 2). Intercept treatment differences will represent treatment differences at baseline (e.g., conditional differences in drinking at baseline) and slope differences will represent changes over time (e.g., did participants in treatment conditions reduce their drinking between baseline and follow-up assessments more than control participants). The intercept includes a random effect, which will model the subject-specific heterogeneity in the alcohol-related outcome thereby controlling for correlated data due to individuals. Main effect models will also control for covariates: age, sexual identity, race, ethnicity, relationship status, and severe interpersonal stigma exposure.

Tests of Mediation and Moderation. Tests of mediation will examine whether perceived drinking norms at the 2-month follow-up mediate relationships between condition and alcohol use outcomes at the 4-month follow-up. PROCESS bootstrap [illegible] measures of potential mediating variables (i.e., norms) and outcomes (i.e. alcohol use, consequences). Moderation analyses will be examined within an MLM

Your response is too large. Try shortening some answers.

use, consequences). Moderation analyses will be examined within an MLM framework and will examine whether the efficacy of treatment PNF varied as a function of participants' baseline drinking, sexual identity, exposure to severe interpersonal stigma, or other demographic characteristics. In the presence of significant interactions, exploratory moderated mediation models [95,97,98] may simultaneously estimate conditional direct and indirect effects associated with the different levels of moderating variables.
Power Analysis. Informed by previous research examining the effects of web-based alcohol PNF on changes in normative perceptions and drinking in other populations (d = .22; [20,99]), the comparably larger effect size revealed in a similar gamified PNF intervention for college students (d = .46; [89]), power analyses using the standard .80 power of detecting a significant effect, $p < .05$, and an effect size of d = .30 indicate a sample size of 375 (125 participants in each condition) to be sufficient to detect small-to-medium effects using repeated measures multi-level models (i.e. 2-levels, 3 arms, randomization at the individual level) as well as tests of mediation and moderation. Thus, our sample size of 500 will allow us to detect modest effects with even 30% attrition.
Data Analytic Plan for Evaluating Feasibility
Descriptive statistics will allow us to assess SMW's level of interest in the LezParlay competition and engagement with the app, (i.e., total number of sign-ups, average number of logins), recruitment origins (e.g., HER app ad, Facebook, Instagram, player referral, etc.), acceptability (mean rating overall and by competition component) and perceived psychological benefits (i.e., proportion of Evaluation Study participants who report benefits). Qualitative text entry responses to items assessing perceived benefits of the LezParlay competition and improvements/features requested for the next version will also be coded by theme/category using a generic inductive qualitative coding approach [100]."

Your response is too large. Try shortening some answers.

12a-i) Imputation techniques to deal with attrition / missing values

Imputation techniques to deal with attrition / missing values: Not all participants will use the intervention/comparator as intended and attrition is typically high in ehealth trials. Specify how participants who did not use the application or dropped out from the trial were treated in the statistical analysis (a complete case analysis is strongly discouraged, and simple imputation techniques such as LOCF may also be problematic [4]).

	1	2	3	4	5	
subitem not at all important	○	○	○	○	◉	essential

Clear selection

Does your paper address subitem 12a-i? *

Copy and paste relevant sections from the manuscript (include quotes in quotation marks "like this" to indicate direct quotes from your manuscript), or elaborate on this item by providing additional information not in the ms, or briefly explain why the item is not applicable/relevant for your study

An intent-to-treat approach will be employed. Multi-level models with full maximum likelihood specification will be used to test aims. This approach makes use of available data for all participants.

12b) Methods for additional analyses, such as subgroup analyses and adjusted analyses

Your response is too large. Try shortening some answers.

Does your paper address CONSORT subitem 12b? *

Copy and paste relevant sections from the manuscript (include quotes in quotation marks "like this" to indicate direct quotes from your manuscript), or elaborate on this item by providing additional information not in the ms, or briefly explain why the item is not applicable/relevant for your study

"Tests of Mediation and Moderation. Tests of mediation will examine whether perceived drinking norms at the 2-month follow-up mediate relationships between condition and alcohol use outcomes at the 4-month follow-up. PROCESS bootstrap tests [95,96] will be used to test mediation. These models will control for baseline measures of potential mediating variables (i.e., norms) and outcomes (i.e. alcohol use, consequences). Moderation analyses will be examined within an MLM framework and will examine whether the efficacy of treatment PNF varied as a function of participants' baseline drinking, sexual identity, exposure to severe interpersonal stigma, or other demographic characteristics. In the presence of significant interactions, exploratory moderated mediation models [95,97,98] may simultaneously estimate conditional direct and indirect effects associated with the different levels of moderating variables."

X26) REB/IRB Approval and Ethical Considerations [recommended as subheading under "Methods"] (not a CONSORT item)

X26-i) Comment on ethics committee approval

	1	2	3	4	5	
subitem not at all important	○	○	○	○	●	essential

Clear selection

Your response is too large. Try shortening some answers.

Does your paper address subitem X26-i?

Copy and paste relevant sections from the manuscript (include quotes in quotation marks "like this" to indicate direct quotes from your manuscript), or elaborate on this item by providing additional information not in the ms, or briefly explain why the item is not applicable/relevant for your study

Yes.
"All recruitment materials, procedures, and intervention materials were approved by the Institutional Review Board at Loyola Marymount University (protocol # LMUIRB2018SU14)."

x26-ii) Outline informed consent procedures

Outline informed consent procedures e.g., if consent was obtained offline or online (how? Checkbox, etc.?), and what information was provided (see 4a-ii). See [6] for some items to be included in informed consent documents.

	1	2	3	4	5	
subitem not at all important	○	○	○	○	◉	essential

Clear selection

Your response is too large. Try shortening some answers.

Does your paper address subitem X26-ii?

Copy and paste relevant sections from the manuscript (include quotes in quotation marks "like this" to indicate direct quotes from your manuscript), or elaborate on this item by providing additional information not in the ms, or briefly explain why the item is not applicable/relevant for your study

Yes.
All recruitment materials linked to LezParlay's informational landing page [87] which presented an overview of the competition and provided a sign-up button that re-directed interested women to view and accept the Terms of Service and Privacy Policy (basic consent for competition participation) before creating an account. All recruitment materials, procedures, and intervention materials were approved by the Institutional Review Board at Loyola Marymount University (protocol # LMUIRB2018SU14).
Procedure

After consenting to take part in the competition, users were prompted to attach a valid mobile phone number to their account and could elect to login with a unique email address and password combination or use their existing Facebook credentials. Next, users created their LezParlay public profile which included a username of their choice and their sexual identity, age-group, relationship status, and pronouns. Users also had the option of uploading a profile photo or Bitmoji to represent them, entering a brief textual self-description, and connecting their Facebook, Twitter, and/or Instagram accounts so that other players could learn about them. Following account creation, players were directed to a home screen which displayed a timer counting down to the close of the current round, as well as buttons to play the current round, browse player profiles, submit and vote on questions to be parlayed in future rounds, view round winners and leaderboards, edit public profile, and change account settings. The specifics of round play and the format of the detailed results (i.e., PNF) delivered at the end of each round are detailed in the Multimedia Appendix.

RCT Sub-Sample Recruitment. There was no upper limit on the number of SMW who could take part in LezParlay and new players were accepted on a rolling basis throughout the competition. We aimed to recruit a minimum of 1,200 LBQ women to sign-up during the first two monthly rounds to ensure that meaningful and stable sexual identity and age-group specific actual norms for drinking and coping behaviors could be delivered in intervention Rounds 3 and 4. From this larger pool of players, 500 drinkers were recruited to take part in a LezParlay Evaluation Study (RCT) during the third month of play. Acting as baseline (T1) for the RCT, Round 3 featured questions about alcohol use, stigma experiences, and a [illegible]

Your response is too large. Try shortening some answers.

submitting answers to alcohol-related questions in Round 3, players were covertly screened for Evaluation Study eligibility based on their answers (i.e., number of

screened for Evaluation Study eligibility based on their answers (i.e., number of drinking days per week and peak drinks on a single day during the past 2 months) as well as their geolocation and number of previous rounds played. Those who played at least 1 previous round, were located in the U.S., and reported drinking alcohol on 3 or more days per week OR having 3 or more drinks on their peak drinking occasion, were invited to take part in the Evaluation Study at the end of the round. Interested potential participants advanced to an informed consent screen which explained that the goal of the study was to evaluate the impact and format of detailed results received in LezParlay and gather player feedback to inform the next version of the competition. Information further detailed that participation in the Evaluation Study simply involved playing and viewing detailed results in subsequent rounds and completing a brief feedback survey at the end of the competition. Participants could earn up to $40 in e-gift cards of their choice for playing subsequent rounds and completing the feedback survey. Those who checked a box indicating that they understood what study participation entailed and desired to participate were welcomed into the study as LezParlay "official testers"."

X26-iii) Safety and security procedures

Safety and security procedures, incl. privacy considerations, and any steps taken to reduce the likelihood or detection of harm (e.g., education and training, availability of a hotline)

	1	2	3	4	5	
subitem not at all important	◉	○	○	○	○	essential

Clear selection

Does your paper address subitem X26-iii?

Copy and paste relevant sections from the manuscript (include quotes in quotation marks "like this" to indicate direct quotes from your manuscript), or elaborate on this item by providing additional information not in the ms, or briefly explain why the item is not applicable/relevant for your study

Consent forms addressed web-app data and user privacy, data usage, risks/benefits. As this was a psychosocial, social-norms-based intervention, harms

Your response is too large. Try shortening some answers.

RESULTS

13a) For each group, the numbers of participants who were randomly assigned, received intended treatment, and were analysed for the primary outcome

NPT: The number of care providers or centers performing the intervention in each group and the number of patients treated by each care provider in each center

Does your paper address CONSORT subitem 13a? *

Copy and paste relevant sections from the manuscript (include quotes in quotation marks "like this" to indicate direct quotes from your manuscript), or elaborate on this item by providing additional information not in the ms, or briefly explain why the item is not applicable/relevant for your study

No. This is a protocol paper submitted prior to any data analysis.

13b) For each group, losses and exclusions after randomisation, together with reasons

Does your paper address CONSORT subitem 13b? (NOTE: Preferably, this is shown in a CONSORT flow diagram) *

Copy and paste relevant sections from the manuscript (include quotes in quotation marks "like this" to indicate direct quotes from your manuscript), or elaborate on this item by providing additional information not in the ms, or briefly explain why the item is not applicable/relevant for your study

No. This is a protocol paper submitted prior to any data analysis.

Your response is too large. Try shortening some answers.

13b-i) Attrition diagram

Strongly recommended: An attrition diagram (e.g., proportion of participants still logging in or using the intervention/comparator in each group plotted over time, similar to a survival curve) or other figures or tables demonstrating usage/dose/engagement.

	1	2	3	4	5	
subitem not at all important	(x)	( )	( )	( )	( )	essential

Clear selection

Does your paper address subitem 13b-i?

Copy and paste relevant sections from the manuscript or cite the figure number if applicable (include quotes in quotation marks "like this" to indicate direct quotes from your manuscript), or elaborate on this item by providing additional information not in the ms, or briefly explain why the item is not applicable/relevant for your study

No. This is a protocol paper submitted prior to any data analysis.

14a) Dates defining the periods of recruitment and follow-up

Your response is too large. Try shortening some answers.

Does your paper address CONSORT subitem 14a? *

Copy and paste relevant sections from the manuscript (include quotes in quotation marks "like this" to indicate direct quotes from your manuscript), or elaborate on this item by providing additional information not in the ms, or briefly explain why the item is not applicable/relevant for your study

Yes.
"Following recent recommendations for testing the real-world feasibility and impact of normative feedback interventions [62,83], LezParlay is examined through a Type I Hybrid-Effectiveness-Implementation Trial [84,85]. That is, in contrast to recruiting LBQ women into a transparent, incentivized, alcohol intervention study, LezParlay was advertised as it would be in the real-world— as a free, online competition designed to test LBQ stereotypes and increase visibility. Only after several rounds of play were a sub-sample of 500 drinkers already taking part in the competition invited to take part in an incentivized Evaluation Study. These players were covertly randomized to receive 1 of 3 unique sequences of feedback (i.e., Alcohol+Coping, Alcohol Only, or Control Only) over 2 consecutive rounds of play. Short-term reductions in norms and drinking were assessed 2 months later organically within the competition through a “Replay Bonus” which invited players to boost their scores by guessing, betting, and reporting on alcohol use and control topics a second time. Then, following the competition, 4 months post-intervention, Evaluation Study participants completed a feedback survey assessing competition acceptability, percieved benefits, and feature requests for the next version of the competition. At the end of this survey, participants reported their alcohol use a final time."

14a-i) Indicate if critical “secular events” fell into the study period

Indicate if critical “secular events” fell into the study period, e.g., significant changes in Internet resources available or “changes in computer hardware or Internet delivery resources”

	1	2	3	4	5	
subitem not at all important	(●)	○	○	○	○	essential

Clear selection

Your response is too large. Try shortening some answers.

Does your paper address subitem 14a-i?

Copy and paste relevant sections from the manuscript (include quotes in quotation marks "like this" to indicate direct quotes from your manuscript), or elaborate on this item by providing additional information not in the ms, or briefly explain why the item is not applicable/relevant for your study

N/A. All participants accessed the web app offsite. It is unknown whether participants faced any interruption in the internet services during the 8 month competition period in which they accessed the web app.

14b) Why the trial ended or was stopped (early)

Does your paper address CONSORT subitem 14b? *

Copy and paste relevant sections from the manuscript (include quotes in quotation marks "like this" to indicate direct quotes from your manuscript), or elaborate on this item by providing additional information not in the ms, or briefly explain why the item is not applicable/relevant for your study

N/A. Not ended/stopped early.

15) A table showing baseline demographic and clinical characteristics for each group

NPT: When applicable, a description of care providers (case volume, qualification, expertise, etc.) and centers (volume) in each group

Does your paper address CONSORT subitem 15? *

Copy and paste relevant sections from the manuscript (include quotes in quotation marks "like this" to indicate direct quotes from your manuscript), or elaborate on this item by providing additional information not in the ms, or briefly explain why the item is not applicable/relevant for your study

No. This is a protocol paper submitted prior to any data analysis.

Your response is too large. Try shortening some answers.

15-i) Report demographics associated with digital divide issues

In ehealth trials it is particularly important to report demographics associated with digital divide issues, such as age, education, gender, social-economic status, computer/Internet/ehealth literacy of the participants, if known.

	1	2	3	4	5	
subitem not at all important	◉	○	○	○	○	essential

Clear selection

Does your paper address subitem 15-i? *

Copy and paste relevant sections from the manuscript (include quotes in quotation marks "like this" to indicate direct quotes from your manuscript), or elaborate on this item by providing additional information not in the ms, or briefly explain why the item is not applicable/relevant for your study

No. This is a protocol paper submitted prior to any data analysis.

16) For each group, number of participants (denominator) included in each analysis and whether the analysis was by original assigned groups

16-i) Report multiple “denominators” and provide definitions

Report multiple “denominators” and provide definitions: Report N’s (and effect sizes) “across a range of study participation [and use] thresholds” [1], e.g., N exposed, N consented, N used more than x times, N used more than y weeks, N participants “used” the intervention/comparator at specific pre-defined time points of interest (in absolute and relative numbers per group). Always clearly define “use” of the intervention.

	1	2	3	4	5	
subitem not at all important	◉	○	○	○	○	essential

Clear selection

Your response is too large. Try shortening some answers.

Does your paper address subitem 16-i? *

Copy and paste relevant sections from the manuscript (include quotes in quotation marks "like this" to indicate direct quotes from your manuscript), or elaborate on this item by providing additional information not in the ms, or briefly explain why the item is not applicable/relevant for your study

N/A. This is a protocol paper submitted prior to any data analysis.

16-ii) Primary analysis should be intent-to-treat

Primary analysis should be intent-to-treat, secondary analyses could include comparing only "users", with the appropriate caveats that this is no longer a randomized sample (see 18-i).

	1	2	3	4	5	
subitem not at all important	○	○	○	○	●	essential

Clear selection

Does your paper address subitem 16-ii?

Copy and paste relevant sections from the manuscript (include quotes in quotation marks "like this" to indicate direct quotes from your manuscript), or elaborate on this item by providing additional information not in the ms, or briefly explain why the item is not applicable/relevant for your study

Yes. this is a protocol paper in which the RCT data analysis plan specifies intent to treat.

17a) For each primary and secondary outcome, results for each group, and the estimated effect size and its precision (such as 95% confidence interval)

Your response is too large. Try shortening some answers.

Does your paper address CONSORT subitem 17a? *

Copy and paste relevant sections from the manuscript (include quotes in quotation marks "like this" to indicate direct quotes from your manuscript), or elaborate on this item by providing additional information not in the ms, or briefly explain why the item is not applicable/relevant for your study

N/A. This is a protocol paper. Trial results are not presented.

17a-i) Presentation of process outcomes such as metrics of use and intensity of use

In addition to primary/secondary (clinical) outcomes, the presentation of process outcomes such as metrics of use and intensity of use (dose, exposure) and their operational definitions is critical. This does not only refer to metrics of attrition (13-b) (often a binary variable), but also to more continuous exposure metrics such as "average session length". These must be accompanied by a technical description how a metric like a "session" is defined (e.g., timeout after idle time) [1] (report under item 6a).

	1	2	3	4	5	
subitem not at all important	◉	○	○	○	○	essential

Clear selection

Does your paper address subitem 17a-i?

Copy and paste relevant sections from the manuscript (include quotes in quotation marks "like this" to indicate direct quotes from your manuscript), or elaborate on this item by providing additional information not in the ms, or briefly explain why the item is not applicable/relevant for your study

This is in no way applicable to this intervention. All participants received identical doses of PNF.

17b) For binary outcomes, presentation of both absolute and relative effect sizes is recommended

Your response is too large. Try shortening some answers.

Does your paper address CONSORT subitem 17b? *

Copy and paste relevant sections from the manuscript (include quotes in quotation marks "like this" to indicate direct quotes from your manuscript), or elaborate on this item by providing additional information not in the ms, or briefly explain why the item is not applicable/relevant for your study

This is a protocol paper. No trial results are presented. Further, no outcomes to be analyzed are binary.

18) Results of any other analyses performed, including subgroup analyses and adjusted analyses, distinguishing pre-specified from exploratory

Does your paper address CONSORT subitem 18? *

Copy and paste relevant sections from the manuscript (include quotes in quotation marks "like this" to indicate direct quotes from your manuscript), or elaborate on this item by providing additional information not in the ms, or briefly explain why the item is not applicable/relevant for your study

This is a protocol paper. No analyses are performed or reported.

18-i) Subgroup analysis of comparing only users

A subgroup analysis of comparing only users is not uncommon in ehealth trials, but if done, it must be stressed that this is a self-selected sample and no longer an unbiased sample from a randomized trial (see 16-iii).

	1	2	3	4	5	
subitem not at all important	(●)	( )	( )	( )	( )	essential

Clear selection

Your response is too large. Try shortening some answers.

Does your paper address subitem 18-i?

Copy and paste relevant sections from the manuscript (include quotes in quotation marks "like this" to indicate direct quotes from your manuscript), or elaborate on this item by providing additional information not in the ms, or briefly explain why the item is not applicable/relevant for your study

This is a protocol paper. No analyses are performed or reported.

19) All important harms or unintended effects in each group

(for specific guidance see CONSORT for harms)

Does your paper address CONSORT subitem 19? *

Copy and paste relevant sections from the manuscript (include quotes in quotation marks "like this" to indicate direct quotes from your manuscript), or elaborate on this item by providing additional information not in the ms, or briefly explain why the item is not applicable/relevant for your study

This is a protocol paper. No analyses are performed or reported.

19-i) Include privacy breaches, technical problems

Include privacy breaches, technical problems. This does not only include physical “harm” to participants, but also incidents such as perceived or real privacy breaches [1], technical problems, and other unexpected/unintended incidents. “Unintended effects” also includes unintended positive effects [2].

	1	2	3	4	5	
subitem not at all important	(●)	( )	( )	( )	( )	essential

Clear selection

Does your paper address subitem 19-i?

Copy and paste relevant sections from the manuscript (include quotes in quotation marks "like this" to indicate direct quotes from your manuscript), or elaborate on this item by providing additional information not in the ms, or briefly explain why the item is not applicable/relevant for your study

Your response is too large. Try shortening some answers.

19-ii) Include qualitative feedback from participants or observations from staff/researchers

Include qualitative feedback from participants or observations from staff/researchers, if available, on strengths and shortcomings of the application, especially if they point to unintended/unexpected effects or uses. This includes (if available) reasons for why people did or did not use the application as intended by the developers.

	1	2	3	4	5	
subitem not at all important	(●)	( )	( )	( )	( )	essential

Clear selection

Does your paper address subitem 19-ii?

Copy and paste relevant sections from the manuscript (include quotes in quotation marks "like this" to indicate direct quotes from your manuscript), or elaborate on this item by providing additional information not in the ms, or briefly explain why the item is not applicable/relevant for your study

This is a protocol paper. No analyses are performed or reported. However, qualitative feedback from participating web app users compose a major component of the feasibility portion of this trial.

DISCUSSION

22) Interpretation consistent with results, balancing benefits and harms, and considering other relevant evidence

NPT: In addition, take into account the choice of the comparator, lack of or partial blinding, and unequal expertise of care providers or centers in each group

Your response is too large. Try shortening some answers.

22-i) Restate study questions and summarize the answers suggested by the data, starting with primary outcomes and process outcomes (use)

Restate study questions and summarize the answers suggested by the data, starting with primary outcomes and process outcomes (use).

	1	2	3	4	5	
subitem not at all important	◉	○	○	○	○	essential

Clear selection

Does your paper address subitem 22-i? *

Copy and paste relevant sections from the manuscript (include quotes in quotation marks "like this" to indicate direct quotes from your manuscript), or elaborate on this item by providing additional information not in the ms, or briefly explain why the item is not applicable/relevant for your study

This is a protocol paper. No analyses are performed or reported.

22-ii) Highlight unanswered new questions, suggest future research

Highlight unanswered new questions, suggest future research.

	1	2	3	4	5	
subitem not at all important	◉	○	○	○	○	essential

Clear selection

Your response is too large. Try shortening some answers.

Does your paper address subitem 22-ii?

Copy and paste relevant sections from the manuscript (include quotes in quotation marks "like this" to indicate direct quotes from your manuscript), or elaborate on this item by providing additional information not in the ms, or briefly explain why the item is not applicable/relevant for your study

Yes, trial limitations are addressed in the discussion.
"Limitations and Future Directions

Funded by the National Institutes of Healths' Exploratory/Developmental R21 grant mechanism, the goal of this initial trial is to evaluate the feasibility and efficacy associated with the LezParlay competition as "minimally viable product" taking the form of an extremely low-cost progressive web app designed and coded by the first author who is a member of the target population. Thus, representing the preliminary step in a larger program of LezParlay gamified PNF intervention research, findings from the present trial will not speak to the feasibility or efficacy of delivering the intervention through a more sophisticated and polished native smartphone application that would likely be more desirable and user-friendly and thus, better-equipped to attract and retain SMW. In addition, PNF is the only intervention component featured in the initial version of LezParlay and further, only static descriptive norms for drinking and coping are corrected. It is possible that findings from this study may suggest high feasibility for the gamified approach and stereotype challenge framing (i.e., large numbers of SMW are engaged by the competition and participants and report psychological benefits) but treatment PNF fails to meaningfully reduce drinkers' consumption and negative alcohol-related consequences relative to control. In this event, the stereotype challenge concept and game mechanics may be retained but future versions of the LezParlay app might expand PNF to correct additional types of alcohol and coping norms (i.e., injunctive, affective, dynamic norms) and/or deliver additional intervention components such as skills training around healthy coping strategies, and/or local alcohol treatment information (i.e., referral to treatment).

Another limitation pertains to the current study's organic assessment of alcohol outcomes and some moderators within rounds of the competition. On one hand, this is a major strength in that it eliminates the demand characteristics that often plague transparent alcohol intervention studies and substantially increased the cost-effectiveness of the trial. However, this also meant that key constructs could only be assessed by a few items, and further, the language of items could not be too formal or clinical in tone. Although the QFM [83] fit well in this regard as a short, validated, measure of alcohol use, it would have also been valuable to include longer validated survey measures to more formally assess alcohol-related [illegible]

Your response is too large. Try shortening some answers.

feasibility and efficacy are demonstrated in the in this initial trial, we anticipate seeking additional funding for a larger trial that will include traditional survey-based [illegible]

seeking additional funding for a larger trial that will include traditional survey-based baseline and follow-up assessments, and test an expanded set of potential mediators and moderators.

A final limitation pertains to this study's narrow focus on the direct impacts of treatment PNF on alcohol-related outcomes. Norms for other health behaviors (i.e., stigma-coping, smoking, exercise, healthcare utilization) were also corrected within the broader competition; however, the RCT was not designed to examine to potential PNF-related changes in these behaviors. Similarly, players were expected to over-estimate a number undesirable, stereotypical behaviors among same-sexual identity peers in non-intervention rounds of the competition (e.g., promiscuity and infidelity among bisexual women, unhealthy relationship behaviors and transphobic attitudes among lesbians). Although outside of the scope of this initial trial, revealing and reinforcing true norms for these experiences and attitudes in LezParlay may carry psychological benefits for SMW partaking in the competition (i.e. reducing identity-related stigma, increasing feelings of belonging, and/or collective self-esteem). Thus, assessing pre-to-post competition changes in these constructs and evaluating the extent to which challenging negative stereotypes through the larger LezParlay competition might buffer stigma-related processes, and thereby reduce drinking and improve other health outcomes, remain critical next steps in the larger program of research."

20) Trial limitations, addressing sources of potential bias, imprecision, and, if relevant, multiplicity of analyses

20-i) Typical limitations in ehealth trials

Typical limitations in ehealth trials: Participants in ehealth trials are rarely blinded. Ehealth trials often look at a multiplicity of outcomes, increasing risk for a Type I error. Discuss biases due to non-use of the intervention/usability issues, biases through informed consent procedures, unexpected events.

	1	2	3	4	5	
subitem not at all important	◉	○	○	○	○	essential

Your response is too large. Try shortening some answers.

Does your paper address subitem 20-i? *

Copy and paste relevant sections from the manuscript (include quotes in quotation marks "like this" to indicate direct quotes from your manuscript), or elaborate on this item by providing additional information not in the ms, or briefly explain why the item is not applicable/relevant for your study

This is an incognito intervention (intervention and control content embedded in a larger web-based activity) wherein participants were blinded. In fact, participants had no awareness that they were even being randomized to a study condition.

21) Generalisability (external validity, applicability) of the trial findings

NPT: External validity of the trial findings according to the intervention, comparators, patients, and care providers or centers involved in the trial

21-i) Generalizability to other populations

Generalizability to other populations: In particular, discuss generalizability to a general Internet population, outside of a RCT setting, and general patient population, including applicability of the study results for other organizations

	1	2	3	4	5	
subitem not at all important	○	○	○	○	○	essential

Your response is too large. Try shortening some answers.

Does your paper address subitem 21-i?

Copy and paste relevant sections from the manuscript (include quotes in quotation marks "like this" to indicate direct quotes from your manuscript), or elaborate on this item by providing additional information not in the ms, or briefly explain why the item is not applicable/relevant for your study

This is not a traditional RCT, rather it is a Type I Implementation-Efficacy Trial. Thus, it is designed to both assess efficacy through an incognito RCT and assess major feasibility questions related to real-world engagement.

"This hybrid trial also follows recent recommendations for the improved design and evaluation of social norms-based health interventions [62], as both mediators and moderators and of intervention effectiveness are examined and importantly, the LezParlay competition is framed and advertised as it would be outside of a study setting. Only later is an incentivized clinical trial sub-sample of drinkers recruited from the larger population of players already engaging with the competition (notably in the absence of traditional study participation incentives). This hybrid design allows the research team to cost-effectively assess the feasibility of drawing large numbers SMW to the broader LezParlay competition via targeted promotional channels, player engagement with different areas of the web app, as well as competition acceptability and ways in which the competition might be improved. Simultaneously, recruiting a sub-sample of alcohol consuming SMW already taking part in the competition into an incognito RCT allows for evaluation of whether PNF on alcohol use and stigma-coping behaviors meaningfully reduce alcohol consumption and negative consequences relative to control PNF. In sum, the current design allows for critical questions about feasibility and efficacy to be jointly addressed with minimal costs to internal or external validity. "

Your response is too large. Try shortening some answers.

21-ii) Discuss if there were elements in the RCT that would be different in a routine application setting

Discuss if there were elements in the RCT that would be different in a routine application setting (e.g., prompts/reminders, more human involvement, training sessions or other co-interventions) and what impact the omission of these elements could have on use, adoption, or outcomes if the intervention is applied outside of a RCT setting.

	1	2	3	4	5	
subitem not at all important	(●)	( )	( )	( )	( )	essential

Clear selection

Does your paper address subitem 21-ii?

Copy and paste relevant sections from the manuscript (include quotes in quotation marks "like this" to indicate direct quotes from your manuscript), or elaborate on this item by providing additional information not in the ms, or briefly explain why the item is not applicable/relevant for your study

See response to 21-i. Same response applies here given the nature of this trial.

OTHER INFORMATION

23) Registration number and name of trial registry

Does your paper address CONSORT subitem 23? *

Copy and paste relevant sections from the manuscript (include quotes in quotation marks "like this" to indicate direct quotes from your manuscript), or elaborate on this item by providing additional information not in the ms, or briefly explain why the item is not applicable/relevant for your study

Yes.
"REGISTRATION: ClinicalTrials.gov NCT03884478;
https://clinicaltrials.gov/ct2/show/NCT03884478"

Your response is too large. Try shortening some answers.

24) Where the full trial protocol can be accessed, if available

Does your paper address CONSORT subitem 24? *

Cite a Multimedia Appendix, other reference, or copy and paste relevant sections from the manuscript (include quotes in quotation marks "like this" to indicate direct quotes from your manuscript), or elaborate on this item by providing additional information not in the ms, or briefly explain why the item is not applicable/relevant for your study

Additional intervention specific information can be found in the MultiMedia Appendix.

25) Sources of funding and other support (such as supply of drugs), role of funders

Does your paper address CONSORT subitem 25? *

Copy and paste relevant sections from the manuscript (include quotes in quotation marks "like this" to indicate direct quotes from your manuscript), or elaborate on this item by providing additional information not in the ms, or briefly explain why the item is not applicable/relevant for your study

Yes.
"FUNDING INFORMATION: This research is supported by grant number R21AA025767-01A1 from the National Institute on Alcohol Abuse and Alcoholism"

X27) Conflicts of Interest (not a CONSORT item)

Your response is too large. Try shortening some answers.

X27-i) State the relation of the study team towards the system being evaluated

In addition to the usual declaration of interests (financial or otherwise), also state the relation of the study team towards the system being evaluated, i.e., state if the authors/evaluators are distinct from or identical with the developers/sponsors of the intervention.

	1	2	3	4	5	
subitem not at all important	○	○	○	○	○	essential

Does your paper address subitem X27-i?

Copy and paste relevant sections from the manuscript (include quotes in quotation marks "like this" to indicate direct quotes from your manuscript), or elaborate on this item by providing additional information not in the ms, or briefly explain why the item is not applicable/relevant for your study

This is noted in the discussion.

About the CONSORT EHEALTH checklist

As a result of using this checklist, did you make changes in your manuscript? *

- ○ yes, major changes
- ◉ yes, minor changes
- ○ no

What were the most important changes you made as a result of using this checklist?

language in title.

Your response is too large. Try shortening some answers.

How much time did you spend on going through the checklist INCLUDING making changes in your manuscript *

4-5 hours. This checklist took forever to complete and perhaps would have been more useful to do ahead of intital submission (prior to paper going out for review and receiving reviewer comments). Many of the items also did not really apply due to the nature of this incognito psychosocial intervention for a non-treatment seeking population AND/OR due to the manuscript in question being a protocol paper rather than a paper reporting findings. Since this is an online survey how about initiating some skip patterns so that authors submitting protocol papers are not responding to items about the reporting of results.

As a result of using this checklist, do you think your manuscript has improved? *

- ○ yes
- ◉ no
- ○ Other:

Would you like to become involved in the CONSORT EHEALTH group?

This would involve for example becoming involved in participating in a workshop and writing an "Explanation and Elaboration" document

- ○ yes
- ○ no
- ◉ Other: I would be happy to contribute in the service of helping to expand/ada

Clear selection

Your response is too large. Try shortening some answers.

Any other comments or questions on CONSORT EHEALTH

This checklist only appears to be appropriate for traditional RCT designs for treatment-seeking populations (despite rather severe limitations related to generalizability and demand characteristics). This checklist should be adapted for hybrid E-Health trials designed to jointly assess efficacy through an incentivized RCT and simultaneously collect feasibility data from other unincentivized sub-groups of participants.

STOP - Save this form as PDF before you click submit

To generate a record that you filled in this form, we recommend to generate a PDF of this page (on a Mac, simply select "print" and then select "print as PDF") before you submit it.

When you submit your (revised) paper to JMIR, please upload the PDF as supplementary file.

Don't worry if some text in the textboxes is cut off, as we still have the complete information in our database. Thank you!

Final step: Click submit !

Click submit so we have your answers in our database!

Submit

Never submit passwords through Google Forms.

This content is neither created nor endorsed by Google. Report Abuse - Terms of Service - Privacy Policy

Google Forms

Your response is too large. Try shortening some answers.
